# Indole-Acrylonitrile Derivatives as Potential Antitumor and Antimicrobial Agents—Synthesis, In Vitro and In Silico Studies

**DOI:** 10.3390/ph16070918

**Published:** 2023-06-22

**Authors:** Anita Kornicka, Karol Gzella, Katarzyna Garbacz, Małgorzata Jarosiewicz, Maria Gdaniec, Joanna Fedorowicz, Łukasz Balewski, Jakub Kokoszka, Anna Ordyszewska

**Affiliations:** 1 Department of Chemical Technology of Drugs, Faculty of Pharmacy, Medical University of Gdansk, 80-416 Gdansk, Poland; karolgzella@gmail.com (K.G.); joanna.fedorowicz@gumed.edu.pl (J.F.); lukasz.balewski@gumed.edu.pl (Ł.B.); kokjaks@gumed.edu.pl (J.K.); 2 Department of Oral Microbiology, Medical Faculty, Medical University of Gdansk, 80-204 Gdansk, Poland; katarzyna.garbacz@gumed.edu.pl (K.G.); malgorzata.jarosiewicz@gumed.edu.pl (M.J.); 3 Faculty of Chemistry, Adam Mickiewicz University, 61-614 Poznań, Poland; maria.gdaniec@amu.edu.pl; 4 Department of Inorganic Chemistry, Faculty of Chemistry and Advanced Materials Centers, Gdańsk University of Technology, Narutowicza 11/12, 80-233 Gdansk, Poland; anna.ordyszewska@pg.edu.pl

**Keywords:** indole-acrylonitrile derivatives, cell growth inhibition, antimicrobial activity, molecular docking, ADME

## Abstract

A series of 2-(1*H*-indol-2-yl)-3-acrylonitrile derivatives, **2a**–**x**, **3**, **4a**–**b**, **5a**–**d**, **6a**–**b**, and **7**, were synthesized as potential antitumor and antimicrobial agents. The structures of the prepared compounds were evaluated based on elemental analysis, IR, ^1^H- and ^13^NMR, as well as MS spectra. X-ray crystal analysis of the representative 2-(1*H*-indol-2-yl)-3-acrylonitrile **2l** showed that the acrylonitrile double bond was *Z*-configured. All compounds were screened at the National Cancer Institute (USA) for their activities against a panel of approximately 60 human tumor cell lines and the relationship between structure and in vitro antitumor activity is discussed. Compounds of interest **2l** and **5a**–**d** showed significant growth inhibition potency against various tumor cell lines with the mean midpoint GI_50_ values of all tests in the range of 0.38–7.91 μM. The prominent compound with remarkable activity (GI_50_ = 0.0244–5.06 μM) and high potency (TGI = 0.0866–0.938 μM) against some cell lines of leukemia (HL-60(TB)), non-small cell lung cancer (NCI-H522), colon cancer (COLO 205), CNS cancer (SF-539, SNB-75), ovarian cancer ((OVCAR-3), renal cancer (A498, RXF 393), and breast cancer (MDA-MB-468) was 3-[4-(dimethylamino)phenyl]-2-(1-methyl-1*H*-indol-2-yl)acrylonitrile (**5c**). Moreover, the selected 2-(1*H*-indol-2-yl)-3-acrylonitriles **2a**–**c** and **2e**–**x** were evaluated for their antibacterial and antifungal activities against Gram-positive and Gram-negative pathogens as well as *Candida albicans*. Among them, 2-(1*H*-indol-2-yl)-3-(1*H*-pyrrol-2-yl)acrylonitrile (**2x**) showed the most potent antimicrobial activity and therefore it can be considered as a lead structure for further development of antimicrobial agents. Finally, molecular docking studies as well as drug-likeness and ADME profile prediction were carried out.

## 1. Introduction

Since the indole motif is a key building block of pharmacologically active natural as well as synthetic molecules, there has been increasing interest in the synthesis and biological research of indole derivatives [1,2,3,4,5]. The importance of the indole skeleton to date has led to the development of diverse bioactive compounds that have been identified with anti-inflammatory [6,7], antioxidant and cytoprotective [8,9], antidepressant [10], anti-migraine [11], antihypertensive [12], antidiabetic [13,14] or antiviral [15] effects and as antitubercular agents [16]. Moreover, indole derivatives represent a significant source of novel antibacterial agents that may exhibit their biological activity through the inhibition of efflux pumps, biofilm or filamentous temperature-sensitive protein Z (FtsZ), and methicillin-resistant *Staphylococcus aureus* pyruvate kinase [17,18,19,20,21,22]. 

Particular attention has been paid to indole-containing compounds with anticancer properties, which exert their activity by affecting numerous biological targets [23,24,25]. For example, the tubulin inhibitors *vincristine* and *vinblastine* isolated from *Catharanthus roseus* are used in the treatment of various cancers [26], whereas *dacomitinib* is a well-known antitumor agent that blocks the activity of epidermal growth factor receptor (EGFR) [27]. Furthermore, indole derivatives have been identified as potent myeloid cell leukemia-1 (Mcl-1) [28] or Pim kinase [29] inhibitors. Antiproliferative effects of the indole-based compounds may also result from the selective inhibition of silent information regular sirtuin 1 (SIRT1) protein, a highly conserved NAD^+^-dependent deacetylase belonging to the sirtuin family [30], as well as σ_2_ receptors, the expression of which is increased in tumor cells with respect to quiescent cells. The σ_2_ receptor ligands can rapidly internalize into tumor cells and induce apoptosis through multiple pathways [31]. On the other hand, an indole-derived histone deacetylase (HDAC) inhibitor, *panobinostat*, has been approved for the treatment of multiple myeloma [32]. Recently, some indole derivatives of ursolic acid have been described as promising DNA topoisomerase II inhibitors with anticancer activity [33].

**Figure 1:**
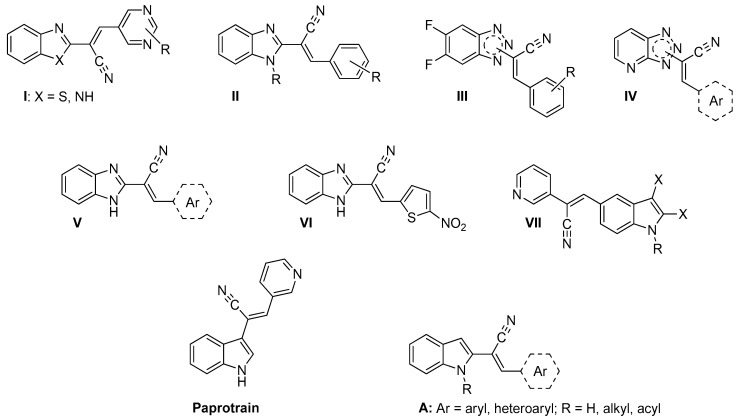
Structures of heteroaryl-acrylonitriles reported and studied in this work.

In addition, 2,3-disubstituted acrylonitriles containing a heteroaromatic core at position 2 of the acrylonitrile moiety have also gained much interest due to their versatile biological activities [34]. These compounds have been shown to possess anti-inflammatory [35], antioxidant [36], antihyperglycemic [37], antiviral [38,39], antimalarial [40] and antimycobacterial [41] properties as well as the ability to inhibit acetylcholinesterase (AChE) activity [42,43]. More recently, some benzazole acrylonitrile-based compounds **I** (Figure 1) were found to be active against both *Escherichia coli* and *Pseudomonas aeruginosa*. The antibacterial activity of these compounds is related to the inhibition of penicillin-binding protein (PBP) and/or *β*-lactamase enzyme [44]. 

Otherwise, several studies have shown the anticancer potential of heteroaryl-acrylonitriles [34]. For example, some acrylonitriles bearing *N*-substituted benzimidazole **II** (Figure 1) [45,46], benzotriazole **III** (Figure 1) [47], or triazolo [4,5-*b*]pyridine **IV** (Figure 1) [48] fragment are able to exert antiproliferative effects against tumor cell lines by interacting with tubulin in the colchicine-binding site. Other heteroaryl-acrylonitrile-based compounds such as 3-aryl-2-(benzimidazol-2-yl)acrylonitriles **V** (Figure 1) have shown significant interaction with ct-DNA, supporting the fact that their antitumor properties could be the consequence of DNA binding [49]. Meanwhile, some of the compounds obtained in our laboratory, such as 3-aryl-2-(1*H*-benzimidazol-2-yl)acrylonitriles, have been identified as potential caspase-9 activators, possessing cytotoxic activity against human cancer cell lines. The most active compound, 2-(benzimidazol-2-yl)-3-(5-nitrothiophen-2-yl)acrylonitrile (**VI**), was on average 10- and 3-fold more potent than *cisplatin* and *etoposide*, respectively, in inhibiting cancer cell growth [50]. In turn, the antiproliferative activity of *N*-alkylindole-substituted 2-(pyrid-3-yl)-acrylonitrile **VII** is probably due to the inhibition of EGFR and VEGFR-2 tyrosine kinases [51], while 2-(indol-3-yl)acrylonitrile (*paprotrain*) and its analogs have been reported as kinesin-like protein 2 (MKLP-2) inhibitors, which could be valuable tools to treat tumors overexpressing MKLP-2 [52,53].

Despite the importance of indole [23] and acrylonitrile [34] scaffolds in the design and discovery of new anticancer agents, indoles substituted at position 2 with the acrylonitrile group have remained unexplored for their biological activities.

In this context, and in connection with a research program on the chemistry and biological activities of 3-aryl-2-heteroaryl-acrylonitriles undertaken in our laboratory years ago [50,54], we considered that 2-(1*H*-indol-2-yl)acrylonitriles of type **A** (Figure 1) may act as potential anticancer agents. These compounds were evaluated for their antimicrobial activity against selected microbial species. In addition, to investigate the importance of the acrylonitrile moiety on biological activity, analogs lacking double bonds were prepared. Furthermore, molecular docking techniques were carried out to rationalize the possible mechanism of action of the most active compounds.

## 2. Results and Discussion

### 2.1. Chemistry

All 2-indolyl-3-acrylonitriles **2a**–**x** were synthesized by the Knoevenagel condensation of 2-(1*H*-indol-2-yl)acetonitrile (**1**) [55] with the appropriate aromatic and heteroaromatic aldehydes. It is worth noting that despite various procedures [56], the Knoevenagel reaction is one of the most useful approaches for the preparation of heteroaryl-acrylonitriles [34,57,58].

**Scheme 1:**
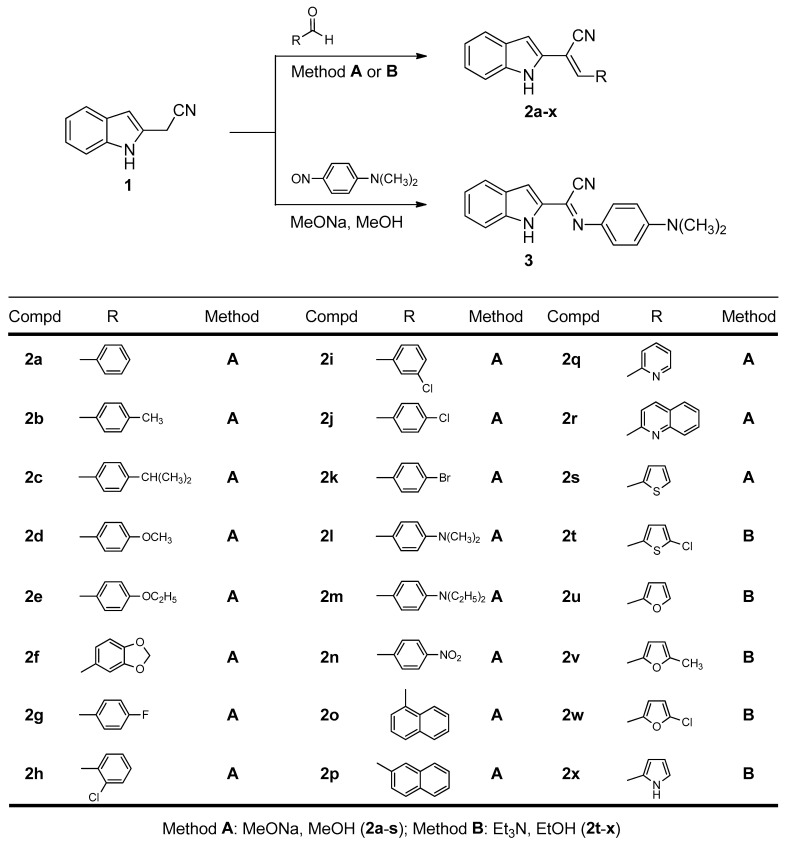
Synthesis of 2-(1*H*-indol-2-yl)-3-acrylonitriles **2a**–**x** and *N*-(4-(dimethylamino)phenyl)-1*H*-indole-2-carbimidoyl cyanide (**3**).

As described in Scheme 1, 2-(1*H*-indol-2-yl)acrylonitriles **2a**–**s** were prepared upon treatment of a methanolic solution of 2-(1*H*-indol-2-yl)acetonitrile (**1**) and aldehydes with sodium methoxide solution at ambient temperature (method A). In turn, 2-(1*H*-indol-2-yl)-3-acrylonitriles **2t**–**x** were synthesized by reacting acetonitrile **1** with the corresponding aldehydes in ethanol at ambient temperature in the presence of triethylamine as a catalyst (method B).

It should be mentioned that 2-(1*H*-indol-2-yl)-3-acrylonitriles **2a** [59,60,61,62,63] and **2b** [61,62,63] have been previously reported. However, there have been no reports of their biological activities.

Compound **1** was then subjected to reaction with *p*-nitrosodimethylaniline in anhydrous methanol in the presence of sodium methoxide to yield the desired iminoacetonitrile **3** (Scheme 1).

The identities of the prepared compounds **2a**–**x** and **3** were confirmed by elemental analysis (C, H, N) as well as spectroscopic data (IR, NMR, and MS) presented in the experimental section (see Section 3).

**Figure 2:**
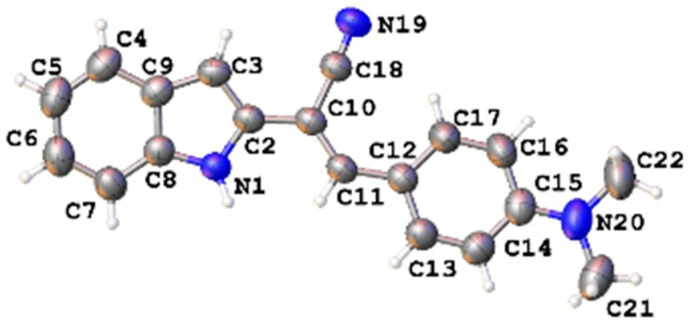
Molecular structure of **2l**. Displacement ellipsoids are shown at the 50% probability level.

It is worth noting that regular NMR methods could not establish the configuration of the acrylonitrile double bond. Therefore, X-ray crystallography was performed on representative 2-(1*H*-indol-2-yl)acrylonitrile **2l**. As shown in Figure 2, in crystal form, compound **2l** adopted a flattened conformation with **a** *Z* configuration at the C10–C11 double bond.

**Scheme 2:**
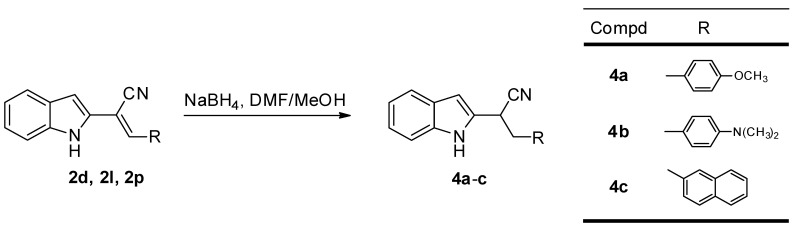
Synthesis of 2-(1*H*-indol-2-yl)-3-phenylpropanenitriles **4a**–**c**.

To determine whether readily available compounds lacking the acrylonitrile double bond would retain any activity of the parent compounds **2d**, **2l**, and **2p**, the 2-(1*H*-indol-2-yl)-3-phenylpropanenitriles **4a**–**c** were synthesized by selective hydrogenation of the olefinic bond using NaBH_4_ in DMF at ambient temperature, according to Scheme 2.

The structures of the compounds **4a**–**c** were confirmed by elemental analysis, IR, NMR, as well as MS spectroscopic data presented in Section 3.

Recently, it has been reported that the introduction of an alkyl or aryl group at position *N*1 of benzimidazole-derived acrylonitriles can result in promising antiproliferative agents [45,46]. Therefore, to explore the structure–activity relationships of the synthesized 2-(1*H*-indol-2-yl)-3-acrylonitriles in more detail, we turned our attention to their *N*-substituted analogs.

**Scheme 3:**
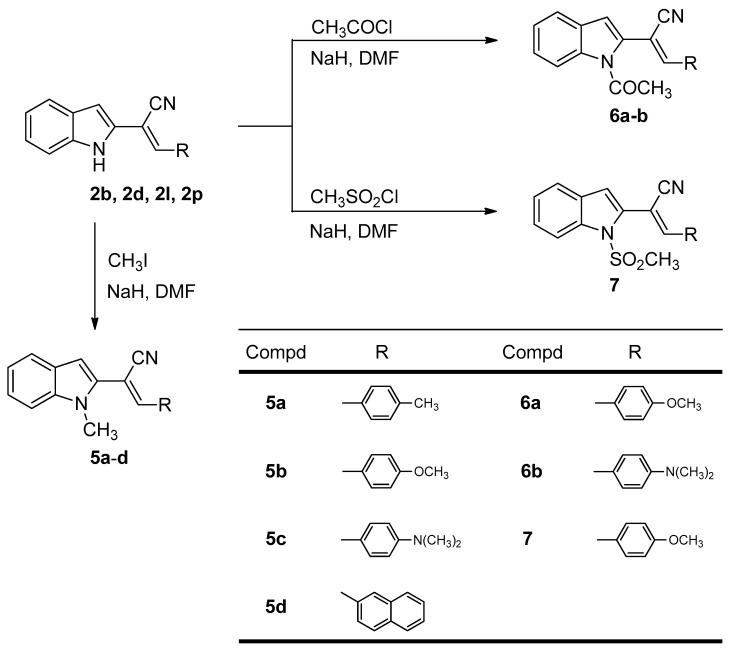
Synthesis of *N*-substituted 2-(1*H*-indol-2-yl)-3-acrylonitriles **5a**–**d**, **6a**–**b**, and **7**.

As depicted in Scheme 3, the reaction procedure leading to the target 1-methyl derivatives **5a**–**d** consisted of the reaction of the corresponding indole-acrylonitrile sodium salts, generated by the treatment of 2-(1*H*-indol-2-yl)-3-acrylonitriles **2b**, **2d**, **2l**, and **2p** with sodium hydride in anhydrous dimethylformamide, with methyl iodide at ambient temperature.

In a similar manner, reaction of 2-(1*H*-indol-2-yl)-3-acrylonitriles **2d** and **2l** with acetyl chloride gave rise to the formation of the corresponding 1-acetyl products **6a** and **6b**, respectively (Scheme 3).

Finally, from reaction of the sodium salt of 2-(1*H*-indol-2-yl)-3-acrylonitrile **2d** with methanesulfonyl chloride, 1-mesyl derivative **7** was isolated (Scheme 3).

The structures of the newly prepared N-substituted 2-(1*H*-indol-2-yl)-3-acrylonitriles **5a**–**d**, **6a**–**b**, and **7** were confirmed by elemental analysis and spectroscopic methods (Section 3).

### 2.2. Biological Evaluation

#### 2.2.1. In Vitro Anticancer Activity

Evaluation of anticancer activity was performed at the National Cancer Institute (NCI, Bethesda, MD, USA), following the known in vitro disease-oriented antitumor screening program against a panel of approximately 60 human cancer cell lines derived from nine cancer types (leukemia, lung, colon, CNS, melanoma, ovarian, renal, prostate, and breast) [64,65,66,67].

Firstly, indole-acrylonitrile derivatives **2a**–**x**, **3**, **4a**–**c**, **5a**–**d**, **6a**–**b**, and **7** were subjected to preliminary screening at a single concentration of 10 μM in approximately 60 cell lines within nine tumor type subpanels. Results for each compounds were reported as a mean graph of the percent growth (%GP) of the treated cells relative to the no-drug control. According to the data analysis of the one-dose mean graphs, it is clear that low mean growth values represented better inhibition activity (%GI = 100 − GP) (values between 0 and 100), while negative values corresponded to lethal activity (values less than 0) (Table 1).

The following can be noted with respect to the mean growth data presented in Table 1 for the tested compounds.

In the series of indole-acrylonitriles **2a**–**p** with aromatic substituent **R** at position 3 of the acrylonitrile moiety, the substituents that gave indisputable anticancer activity were 4-methoxyphenyl (compound **2d**), 4-(dimethylamino)phenyl (compound **2l**), and 2-naphthyl (compound **2p**).

The incorporation of the heteroaromatic ring as substituent **R** at position 3 of the acrylonitrile moiety afforded compounds **2q**–**x** with weak activity.

Replacement of the acrylonitrile moiety by imino-acetonitrile led to a dramatic decrease in activity (comparing compounds **2l** and **3**). Additionally, hydrogenation of the acrylonitrile double bond was associated with very poor activity (compounds **4a**–**c**).

The introduction of a methyl substituent (**5a**–**d**) into position 1 of the indole ring of the parent structure led to compounds with greater or equivalent activity compared with their acrylonitrile counterparts **2b**, **2d**, **2l**, and **2p**. Interestingly, the introduction of an acetyl group into position 1 of the indole ring of the acrylonitrile analogs had a more varied effect on activity than the introduction of a methyl group (comparing compounds **2b** with **6a**, and **2l** with **6b**). Moreover, a decrease in activity was observed when a methylsulfonyl group was attached to the nitrogen atom of the indole scaffold (comparing compounds **2l** and **7**).

From the pattern of the mean growth graph it was apparent that compounds **2l** and **5a**–**d** exerted significant growth inhibition against various cancer cell lines representing different cancer types. Therefore, these compounds were selected by NCI for a secondary screening at five concentration levels (0.01–100 μM).

**Figure 3:**
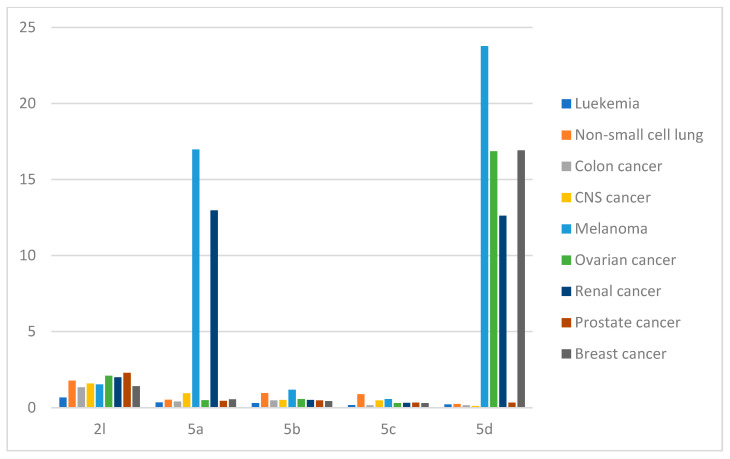
Comparison of the mean GI_50_ values per panel displayed by compounds **2l** and **5a**–**d**.

Data for the selected indole-acrylonitrile derivatives **2l** and **5a**–**d** are recorded in Table 2 and Table 3 and Figure 3. The antitumor activity of the tested compounds is reported for each cell line by GI_50_ value (GI_50_ = molar concentration of the compound that inhibits 50% net cell growth) and TGI value (TGI = molar concentration of the compound leading to total inhibition). Furthermore, a mean graph midpoint (MG-MID) is depicted for the GI_50_ parameter, giving the averaged activity parameter over all cell lines.

As shown in Table 2, indole-acrylonitrile **2l** bearing 4-(dimethylamino)phenyl as substituent **R** was characterized by relatively high inhibitory activity, with GI_50_ values in the range of 0.228–6.6 μM. In addition, derivative **2l** was found to exert a significant cytostatic effect against some cell lines of leukemia (CCRF-CEM, RPMI-8226), lung cancer (HOP-62, HOP-92), colon cancer (COLO 205, HT29), CNS cancer (SF-295, SNB-75, U251), and melanoma (MDA-MB-435), with TGI values between 0.641 and 18.6 μM. In regard to the effect in the entire panel of tumor cell lines, compound **2l** demonstrated pronounced antiproliferative activity with GI_50_ MG-MID values ranging from 0.66 to 2.28 μM, especially against leukemia cells (GI_50_ MG-MID = 0.66 μM) (Table 3, Figure 3).

As expected, *N*-methyl-substituted analogue **5c** showed improved antitumor properties in comparison with its indole-acrylonitrile counterpart **2l** (GI_50_ = 0.0193–5.06 μM vs. 0.228–6.6 μM) (Table 2). In addition, compound **5c** exhibited remarkable cytostatic activity at low TGI level < 5.3 μM towards 12 cancer cell lines, being particularly effective against 9 various cell lines out of 7 subpanels with TGI values in the submicromolar range of 0.0866–0.938 μM. It was observed that non-small cell lung cancer cell line NCI-H522 was the most susceptible cell line with TGI = 0.0866 μM (Table 2). On the other hand, the highest overall sensitivity to this compound was found for the subpanels of leukemia and colon cancer cell lines, for which GI_50_ MG-MID values were 0.17 and 0.15 μM, respectively (Table 3, Figure 3). Notably, compared with its counterpart **2l**, derivative **5c** was 3–9-fold more potent in individual subpanels of cancer cell lines (GI_50_ MG-MID = 0.15–0.56 μM vs. GI_50_ MG-MID = 0.66–2.28 μM) (Table 3, Figure 3).

Moreover, *N*-methyl-substituted compound **5b** with 4-methoxyphenyl as substituent **R** also demonstrated significant antitumor activity against all of the tested cell lines, with GI_50_ values ranging from 0.153 to 6.96 μM (Table 2). It is worth noting that derivative **5b** acted as a potent inhibitor against 18 human tumor cell lines of 8 subpanels, with calculated TGI values in the range of 0.403–21.3 μM. Submicromolar TGI values were found for HL-60(TB) and SR leukemia (TGI = 0.582 and 0.94 μM, respectively), NCI-H522 non-small cell lung cancer (TGI = 0.554 μM), SNB-75 CNS cancer (TGI = 0.514 μM), MDM-MB-435 melanoma (TGI = 0.403 μM), OVCAR-3 ovarian cancer (TGI = 0.955 μM), A498 and RXF 393 renal cancer (TGI = 0.867 and 0.751 μM, respectively), and MDA-MB-468 breast cancer (TGI = 0.826 μM) cell lines (Table 2). Nevertheless, concerning overall activity, it should be noted that compound **5b** proved to be slightly less potent than derivative **5c** (GI_50_ MG-MID = 0.29–1.17 μM vs. 0.15–0.56 μM) (Table 3, Figure 3). A further decrease in potency was observed when 4-(dimethylamino)phenyl as substituent **R** in compound **5c** was replaced with 4-methylphenyl moiety in analogue **5a** (GI_50_ = 0.0193–5.06 μM vs.0.0726–100 μM and GI_50_ MG-MID = 0.15–0.56 μM vs. 0.35–16.98 μM) (Table 2 and Table 3, Figure 3). However, the latter derivative still retained pronounced growth inhibitory properties especially against certain cell lines, including HL-60(TB) leukemia, NCI-H522 non-small cell lung cancer, SF-295 and SF-539 CNS cancer, MDA-MB-435 melanoma, OVCAR-3 ovarian cancer, A498 renal cancer, and BT-549 breast cancer, with TGI values in the range of 0.254–7.12 μM (Table 2).

Another modification of **5c**, consisting of replacement of 4-(dimethylamino)phenyl as substituent **R** at position 3 of the acrylonitrile moiety with a 2-naphthyl group, resulted in compound **5d**, which generally exhibited higher activity than **5c** against cell lines from the non-small cell lung cancer and CNS cancer subpanels (GI_50_ MG-MID = 0.24 and 0.11 μM vs. 0.89 and 0.47 μM, respectively) (Table 3, Figure 3). In addition, compound **5d** acted selectively as a potent inhibitor against HL-60(TB) leukemia (GI_50_ = 0.179 μM, TGI = 0.703 μM), SF-539 CNS cancer (GI_50_ = 0.0613 μM, TGI = 0.552 μM), MDA-MB-435 melanoma (GI_50_ = 0.0318 μM, TGI = 0.103 μM), and A498 renal cancer (GI_50_ = 0.03 μM, TGI = 0.418 μM) cell lines (Table 2). On the other hand, taking into account overall potency, compound **5d** proved to be the least active in this series (full panel GI_50_ MG-MID > 7.91 μM) (Table 3).

From these results it was concluded that the combination of the *N*-methyl group in the indole ring with the 4-dimethylaminophenyl group at position 3 of the acrylonitrile moiety resulted in derivative **5c** with optimal properties (full panel GI_50_ MD-MIG = 0.38 μM vs. 0.60–7.91 μM) (Table 3).

#### 2.2.2. Antimicrobial Activity against Reference Microbial Strains

The synthesized indole-acrylonitriles **2a**–**c** and **2e**–**x** were evaluated for their in vitro antimicrobial activity against Gram-positive bacteria (MSSA *Staphylococcus aureus* ATCC 6538, American Type Culture Collection, USA, *Staphylococcus epidermis* PMC 2118, Polish Collection of Microorganisms, Poland, *Enterococcus faecalis* ATCC 11420), Gram-negative bacteria (*Escherichia coli* ATCC 11229, *Pseudomonas aeruginosa* ATCC 15442), as well as a fungal species (*Candida albicans* ATCC 10231). The tests were performed using a serial dilution method, allowing the determination of the minimal concentration inhibiting bacterial growth (MIC), minimal bactericidal concentration (MBC), and minimal fungicidal concentration (MFC). The obtained results are presented in Table 4.

Most of the investigated compounds showed no or negligible antimicrobial activity; their MIC, MBC, and MFC values were between ≥128 and ≥256 μg/mL. On the other hand, in the series of indole-acrylonitriles **2a**–**p** with aromatic substituent **R** in position 3 of the acrylonitrile moiety, 3-chlorophenyl derivative **2i** was characterized by relatively high antibacterial activity against Gram-positive bacteria *S. aureus* ATCC 6538 and *S. epidermis* PMC 2118, with MIC and MBC values ranging from 8 to 16 μg/mL (Table 4). Interestingly, replacement of the 3-chlorophenyl group in **2i** for either 2-chlorophenyl (compound **2h**) or 4-chlorophenyl (compound **2j**) resulted in a severe reduction in activity (MIC = 128–256 μg/mL, MBC/MFC ≥ 256 μg/mL, Table 4). In turn, indole-acrylonitrile **2n** bearing 4-nitrophenyl as substituent **R** exhibited moderate bacteriostatic activity against *S. aureus* ATCC 6538 (MIC = 64 μg/mL), while its bactericidal effect against this strain was weak (MBC = 128 μg/mL, Table 4).

In the series of heteroaromatic analogs **2q**–**x**, promising antimicrobial activity against some bacterial species was demonstrated by compounds **2q**, **2s**, and **2x** (Table 4). Thus, derivative **2q** containing pyridine as substituent **R** in position 3 of the acrylonitrile moiety was found to have pronounced potential against two bacterial Gram-positive strains: *S. aureus* ATCC 6538 and *S. epidermis* PMC 2118. The MIC and MBC values obtained for this compound against both strains were 8 and 16 μg/mL, respectively (Table 4). Changing the pyridine ring of **2q** to a thiazole (compound **2s**) led to a slight decrease in activity against the same bacterial strains (MIC = 16–32 μg/mL, MBC = 32 μg/mL) (Table 4). Otherwise, thiazole derivative **2s** displayed significant antifungal activity against *C. albicans* with an MIC value of 16 μg/mL (Table 4). However, the highest activity among all the tested compounds was exhibited by 3-pyrole derivative **2x**. Thus, compound **2x** displayed relatively high antibacterial potency against all Gram-positive bacteria tested, with MIC values in the range of 8–32 μg/mL and MBC values of 32 μg/mL. Furthermore, it was the only compound that was found to be effective against Gram-negative bacteria, presenting antibacterial activity against *E. coli* with MIC and MBC values of 32 μg/mL. In addition, 3-pyrole derivative **2x** was characterized by great antifungal activity against *C. albicans*, with MIC and MFC values of 4 and 8 μg/mL, respectively (Table 4).

It is notable that despite testing a large number of compounds, it was not possible to derive a relationship between structure and activity in the studied series of indole-acrylonitrile derivatives **2a**–**c** and **2e**–**x**. On the other hand, compounds **2i**, **2q**, **2s**, and **2x** with interesting antimicrobial activity did not exhibit antitumor effects against the tested cancer cell lines.

#### 2.2.3. Antibacterial Activity against Clinical Staphylococcus Aureus Strains

The most pronounced compounds **2i**, **2q**, **2s**, and **2x** were further evaluated for their bacteriostatic and bactericidal activities against a panel of clinical isolates of *Staphylococcus aureus* (79, 124, 128, 143, 177, 220 and 244) derived from various human infections. The MIC and MBC values of the tested compounds are shown in Table 5.

As revealed by the data in Table 5, indole-acrylonitriles **2i**, **2q**, and **2s** were inactive against the tested *Staphylococcus aureus* strains isolated from clinical specimens; their MIC and MBC values were between >128 and >256 μg/mL. On the other hand, satisfactory MIC and MBC values were obtained for compound **2x**. It was shown that this compound had ability to inhibit the growth of the clinical isolates at a low concentration of 16 μg/mL. In addition, indole-acrylonitrile **2x** was characterized by relatively strong or moderate bactericidal activity, with MBC values of 32 and 64 μg/mL.

Further studies also indicated relatively high and moderate bactericidal activities of compound **2x** against both clinical methicillin-resistant and -sensitive *Staphylococcus aureus* strains (MRSA 1–5, MSSA 6–10), with MIC and MBC values of 16 and 64 μg/mL, respectively (Table 6). It should be noted that both MRSA and MSSA strains exhibited similar sensitivity to the tested compound **2x**, while MRSA strains are generally more resistant to antibiotics and antimicrobial compounds than MSSA strains [68].

### 2.3. Docking Studies

#### 2.3.1. Docking to Anticancer Targets

In order to rationalize the experimentally assessed antiproliferative properties of the synthesized compounds against cancer cell lines, computational analysis was undertaken. As mentioned above, heteroaryl-acrylonitriles can exhibit antiproliferative effects by inhibiting tubulin polymerization due to their ability to bind to the colchicine-binding site [45,46,47,48]. Although some synthetic compounds that bind to the colchicine site have been evaluated in clinical trials [69], none have been approved for cancer therapy to date. Therefore, this binding site still offers challenging opportunities for drug development [70].

The caspase signaling pathway has also generated considerable attention as a promising cancer therapeutic strategy [71]. Previously our research group identified a series of 3-aryl-2-(1*H*-benzimidazol-2-yl)acrylonitriles as potential caspase-3 and -9 activators with cancer cell growth inhibitory properties [50]. On the basis of these results, we considered the induction of the activity of apoptotic enzymes such as caspase-3 and -9.

With the above in mind, molecular docking studies of the obtained series of compounds were performed in the binding pockets of the following proteins: caspase-3, caspase-9, and tubulin (PDB codes: 2xyp [72], 2ar9 [73], and 5eyp [74], respectively).

From the obtained FRED Chemgauss4 scores (Table S1, Supplementary Materials), it was concluded that most of the proposed ligands exhibited significant affinity to caspase-3, including the active derivatives **2l**, **5c**, and **5d** (Chemgauss4 scores ranging from −5.32 to −6.09 for the top ranked poses). Furthermore, the active compounds **2l** and **5d** were ranked relatively high for the caspase-9 binding pocket (Chemgauss4 scores of −3.57 and −3.05, respectively). On the other hand, the potent analogs **5a**–**c** as well as **5d** were characterized by relatively high affinity for the tubulin binding pocket (Chemgauss4 scores ranging from −11.53 to −13.30).

**Figure 4:**
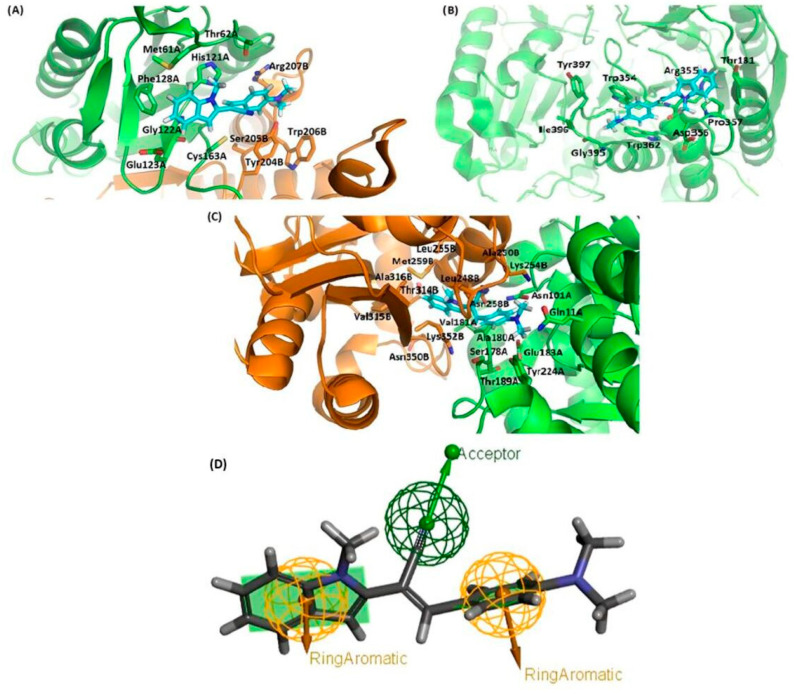
The highest-scored poses of compound **5c** (cyan sticks) docked in the active site of: caspase-3 (p17 and p12 subunits in green and orange, respectively), (**A**), caspase-9 (**B**), and tubulin (α- and β-tubulin in green and orange, respectively), (**C**); graphic representation of compound **5c** fitted into the ligand-based pharmacophore (**D**). Hydrogen bonds are indicated as black dotted lines. For clarity, only relevant amino acids are presented. (**A**–**C**) were prepared using PyMOL 1.5.0.3., and (**D**) was generated by BIOVA Discovery Studio Visualizer (for interpretation of the references to color in this figure legend, the reader is referred to the Web version of this article).

The highest ranked poses of the most potent compound **5c** docked in the target proteins are presented in Figure 4, while 2D diagrams of the interactions of the active derivatives **2l** and **5a**–**d** can be found in the Supplementary Materials (Tables S2–S4).

As shown in Figure 4A, in the binding site of caspase-3, the aromatic rings of ligand **5c** made hydrophobic contacts with the Met61 and Cys163 residues present in the p17 subunit as well as Arg207 in the p12 subunit. Side chains of His121 and Tyr204 from p17 and p12, respectively, formed π-π stacking interactions with the ligand core. Van der Waals forces were created with Thr64, Gly122, Glu123, and Phe128 in p17 as well as Tyr204, Ser205, Trp206, and Arg207 in p12.

In the case of caspase-9 (Figure 4B), van der Waals interactions were formed between ligand **5c** and Thr181, Asp356, Trp362, Gly395, Ile396, and Tyr397. The NH group from the main chain of Arg355 formed a hydrogen bond with a length of 2.6 A with a nitrile nitrogen atom of **5c**. Hydrophobic contacts between the ligand and Trp354 as well as Pro357 were also found.

According to Figure 4C, ligand **5c** created van der Waals interactions with Gln11, Asn101, Ser178, Thr179, Tyr224, and Asn249 from α-tubulin as well as Leu248, Ala250, Lys254, Leu255, Asn258, Thr314, Val315, and Asn350 from *β*-tubulin in the colchicine-binding site of tubulin. Hydrophobic contacts were formed with Ala180 and Val181 from α-tubulin as well as Met259, Ala316, and Lys352 from *β*-tubulin. Glu183 from α-tubulin interacted via anion-π contact.

Based on the above results, it was concluded that the most important features of the pharmacophore were the two aromatic rings separated by two carbon atoms connected via double bound along with the nitrile moiety, which served as a hydrogen bond acceptor, as shown in Figure 4D. Bulky substituents were not allowed on the indole nitrogen atom, since only compounds without a substituent (**2l**) or bearing a methyl group (**5a**–**d**) presented pronounced activity. For a more beneficial effect, the additional pendant aromatic ring should be 2-naphthyl or *para*-substituted phenyl. Especially advantageous was the introduction of a dimethylamine moiety, which was consistent with literature data [45,46].

#### 2.3.2. Docking to Antibacterial Targets

The antibacterial activity of the acrylonitrile-based compounds could be potentially associated with their affinity to bacterial enzymes involved in the synthesis of peptidoglycan, which is the major component of bacterial cell walls, i.e., penicillin-binding protein 4 (PBP4) and/or *β*-lactamase [44]. Thus, the novel ligands were docked in the active sites of the aforementioned proteins from *E. coli* (PDB codes 2ex8 [75] and 1fqg [76], respectively).

**Figure 5:**
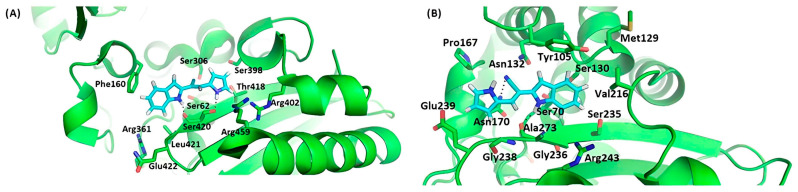
The highest-scored poses of compound **2x** (cyan sticks) docked in the active site of *E. coli* enzyme (green), PBP4 (**A**), and *β*-lactamase (**B**). Hydrogen bonds are indicated as black dotted lines. For clarity, only relevant amino acids are presented. The figure was prepared using PyMOL 1.5.0.3. (For interpretation of the references to color in this figure legend, the reader is referred to the Web version of this article).

As revealed by the FRED Chemgauss4 scores (Table S1, Supplementary Materials), some of the ligands were ranked higher than the original ligand in the crystal structures, penicillin G. For example, the active derivatives **2i** and **2x** were bound more strongly in the PBP4 active site than benzylpenicillin (−5.90 and −6.00 vs. −5.66, respectively). In the β-lactamase-binding pocket, the ligand **2i** also achieved a greater score than the original ligand (−9.63 vs. −9.55), while the most potent ligand **2x** was ranked lower than penicillin G with Chemgauss4 score of −7.75. The molecular structure of the most active derivative **2x** docked in the active pockets of the analyzed proteins is presented in Figure 5, while the 2D diagrams of interactions are included in Supplementary Materials (Tables S5 and S6).

Within PBP4 (Figure 5A), van der Waals interactions were formed between ligand **2x** and Ser62, Phe160, Ser306, Arg361, Ser398, Arg402, Thr418, Leu421, Gln422, and Arg459. The indole NH group of the ligand created a hydrogen bond with a length of 1.6 A with the oxygen atom of the carboxyl group from the main chain of Ser420. However, the hydroxyl group present in the side chain of this amino acid residue formed an unfavorable hydrogen donor–donor type interaction with the NH group of the pyrrole ring present within the ligand structure (length of 1.7 A).

In the binding site of *β*-lactamase (Figure 5B), Ala237 formed hydrophobic contacts with the indole ring of the derivative **2x**. The main chain of this amino acid formed a hydrogen bond of 2.2 A with the NH group of the indole ring. Another hydrogen bond was created between the nitrile nitrogen atom and the Asp170 residue (3.0 A). Van der Waals forces were detected with Ser70, Tyr105, Met129, Ser130, Asn132, Pro167, Val216, Ser235, Gly236, Gly238, Glu239, and Arg243.

### 2.4. In Silico Physicochemical, Pharmacokinetic and Drug-Likeness Predictions

**Figure 6:**
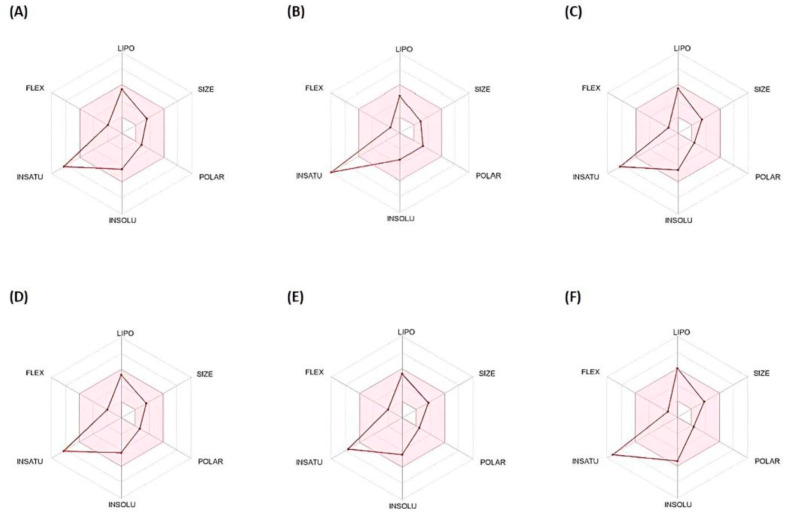
Oral bioavailability radar charts for the studied compounds **2l**, **2x**, and **5a**–**d**. In bioavailability radar, the pink area represents the optimal range for each physicochemical property of oral bioavailability (LIPO—lipophilicity, SIZE—size, POLAR—polarity, INSOLU—solubility, INSATU—saturation and FLEX—flexibility), while the red lines represent compounds: (**A**) **2l**, (**B**) **2x**, (**C**) **5a**, (**D**) **5b**, (**E**) **5c**, and (**F**) **5d**.

**Figure 7:**
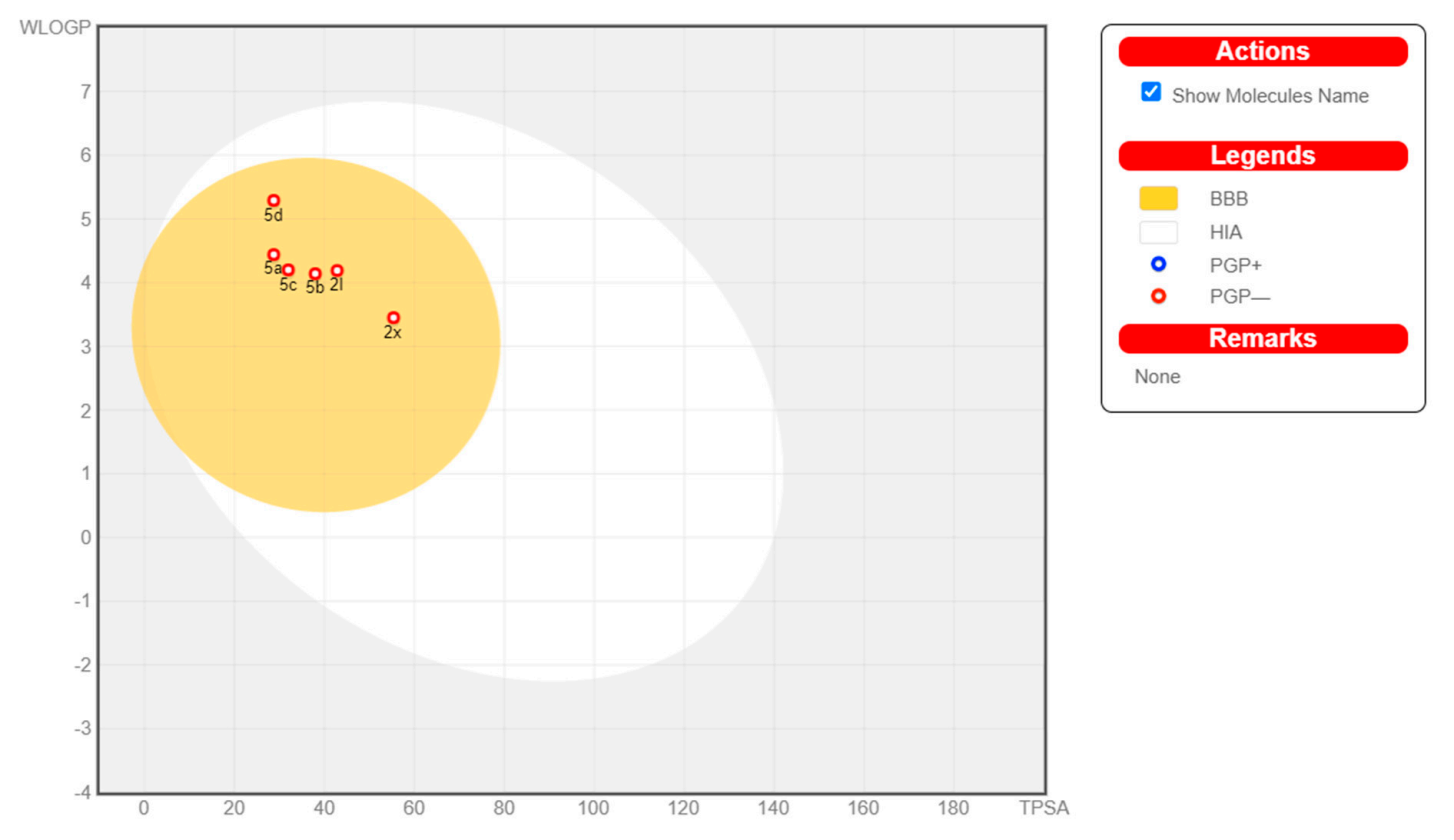
BOILED-Egg plot for the studied compounds **2l**, **2x**, and **5a**–**d**.

The free available SwissADME web tool (http://www.swissadme.ch (accessed on 9 May 2023)) accessed on 16 February 2023 was employed to evaluate the physicochemical characteristics and predict the pharmacokinetic and drug-likeness properties of the most potent 2-(1*H*-indol-2-yl)-3-acrylonitriles **2l**, **2x,** and **5a**–**d** [77]. The results are presented in Table 7 and Figure 6 and Figure 7 (see Table S7 in the Supplementary Materials for more details).

As can be seen from the data in Table 7, the tested molecules were characterized by reasonable polarity (TPSA values in the range of 28.78–55.37 Å^2^) and suitable lipophilicity (ClogP values ranging from 3.49 to 4.41), so they were expected to be soluble or moderately soluble in water.

Moreover, according to Table 7, the bioavailable radar charts in Figure 6, and the BOILED-Egg plot in Figure 7, the investigated compounds were predicted to possess high gastrointestinal tract (GI) absorption and blood–brain barrier (BBB) permeability. In this regard, all of the tested molecules showed the same bioavailability score of 0.55, which suggested desirable pharmacokinetic properties (Table 7). Additionally, as shown in Table 7, compounds **2l**, **2x**, and **5a**–**d** met all the criteria according to Lipinski’s “rule of five” as one of the key drug-likeness characteristics [78].

Other drug-likeness predictions, namely Caco-2 cell and MDCK cell permeabilities, were calculated using the PreADMET online server (http://preadmet.bmdrc.kr (accessed on 9 May 2023)) accessed on 1 June 2023. The in vitro Caco-2 cell permeability results classified indole-acrylonitriles **2l**, **2x**, and **5a**–**d** as moderate permeability compounds (12.20–57.65 nm/s), with **2x** possessing the worst predicted permeability (12.20 nm/s). On the other hand, the in vitro MDCK permeability was more varied: moderate permeability was obtained for compounds **2x**, **5a**, and **5d** (46.73–54.34 nm/s), compound **5b** showed low permeability (14.80 nm/s), while analogs **2l** and **5c** bearing the dimethylamino moiety could be characterized as poorly permeable compounds (0.09 and 0.16 nm/s, respectively) (Table S8 in Supplementary Materials).

## 3. Materials and Methods

### 3.1. Chemistry

#### 3.1.1. General Information

Melting points were measured using a Boetius apparatus (VEB Analytik Dresden, Germany) and are uncorrected. IR spectra were obtained in KBr pellets using a Nicolet 380 FTIR 1600 spectrometer. Magnetic resonance spectra (NMR) (Agilent, Karlsruhe, Germany) were recorded using a Varian Mercury-VX 300 or Bruker Avance III HD 400 spectrometer. ^1^H and ^13^C chemical shifts (δ) are reported in ppm relative to the residual solvent signals at 2.50 and 39.5 ppm (DMSO-*d_6_*). Coupling constants (*J*) are given in hertz (Hz). The mass spectra were recorded on a Shimadzu LCMS-2010 EV (Tokyo, Japan) spectrometer equipped with an electrospray source. The ESI-MS spectra were registered in positive- or negative-ion mode.

Diffraction data for **2l** were collected at room temperature using an Oxford Diffraction SuperNova diffractometer (Agilent Technologies Inc., Santa Clara, CA, USA) with Cu Kα radiation and processed using CrysAlisPro software version 1.171.33.48 [79]. The structure was solved using the program SHELXT [80] and refined using the full-matrix least-squares method on *F*^2^ with SHELXL-2018/3 [81] with Olex2 software version 1.5 [82].

Preparative thin-layer chromatography was performed on silica gel 60 PF254 containing gypsum (Merck KGaA, Darmstadt, FRG) with the aid of a Chromatotron^®^ using the reported solvent systems. 2-(1*H*-Indol-2-yl)acetonitrile (1) was obtained according to the published method [55].

#### 3.1.2. General Procedure for the Preparation of 2-(1H-Indol-2-yl)-3-acrylonitriles **2a**–**s**

To a solution of 2-(1*H*-indol-2-yl)acetonitrile (**1**) (312 mg, 2.0 mmol) in anhydrous methanol (10 mL) was added dropwise a solution of sodium methoxide (60 mg of sodium in 6 mL of anhydrous methanol). The reaction mixture was stirred for 30 min, and then the appropriate aldehyde was added (4.0 mmol). After stirring overnight at ambient temperature, the product that precipitated was collected by vacuum filtration, washed with methanol, and if necessary subjected to silica gel column chromatography with dichloromethane as the eluent. In this manner, the following compounds were obtained.

*2-(1H-Indol-2-yl)-3-phenylacrylonitrile* (**2a**). Yield: 39% (yellow solid); m.p. 197–199 °C (m.p. 173–174 °C [63]); IR ν_max_ (KBr, cm^−1^): 3360, 3085, 3058, 3023, 2224, 1433, 1341, 1302, 914, 782, 726, 676; ^1^H NMR (300 MHz, DMSO-*d_6_*) δ (ppm): 11.74 (s, 1H, NH), 7.92 (s, 1H, CH), 7.87 (d, *J* = 7.1 Hz, 2H, 2 × C-H_arom_), 7.62–7.45 (m, 4H, 4 × C-H_arom_), 7.41 (d, *J* = 8.9 Hz, 1H, C-H_arom_), 7.18 (t, *J* = 7.1 Hz, 1H, C-H_arom_), 7.04 (t, *J* = 7.5 Hz, 1H, C-H_arom_), 6.80 (s, 1H, C_3_-H, indole); ^13^C NMR (75 MHz, DMSO-*d_6_*) δ (ppm): 138.6, 138.2, 134.0, 133.7, 130.9, 129.6 (two overlapping signals), 129.2 (two overlapping signals), 128.2, 123.9, 121.3, 120.4, 117.5, 111.8, 104.2, 103.7; MS (ESI) *m*/*z*: 243 [M − H]^−^. Anal. calcd for C_17_H_12_N_2_ (244.29) (%): C, 83.58; H, 4.95; N, 11.47. Found: C, 83.32; H, 5.01; N, 11.67.
*2-(1H-Indol-2-yl)-3-(p-tolyl)acrylonitrile* (**2b**). Yield: 62% (yellow solid); m.p. 223–224 °C (192–194 °C [63]); IR ν_max_ (KBr, cm^−1^): 3356, 3045, 2918, 2223, 1611, 1435, 1302, 898, 798, 782, 730, 607; ^1^H NMR (400 MHz, DMSO-*d_6_*) δ (ppm): 11.72 (s, 1H, NH), 7.90 (s, 1H, CH), 7.80 (d, *J* = 8.2 Hz, 2H, 2 × C-H_arom_), 7.60 (d, *J* = 7.9 Hz, 1H, C-H_arom_), 7.43–7.37 (m, 1H, C-H_arom_), 7.38 (d, *J* = 8.0 Hz, 2H, 2 × C-H_arom_), 7.20 (t, *J* = 7.0 Hz, 1H, C-H_arom_), 7.05 (t, *J* = 7.0 Hz, 1H, C-H_arom_), 6.79 (s, 1H, C_3_-H, indole), 2.39 (s, 3H, CH_3_); MS (ESI) *m*/*z*: 257 [M − H]^−^. Anal. calcd for C_18_H_14_N_2_ (258.32) (%): C, 83.69; H, 5.46; N, 10.84. Found: C, 83.57; H, 5.25; N, 11.18.
*2-(1H-Indol-2-yl)-3-(4-isopropylphenyl)acrylonitrile* (**2c**). Yield: 21% (yellow solid); m.p. 176–178 °C; IR ν_max_ (KBr, cm^−1^): 3327, 2956, 2869, 2233, 1606, 1417, 1302, 897, 827, 784, 747, 730, 613; ^1^H NMR (400 MHz, DMSO-*d_6_*) δ (ppm): 11.74 (s, 1H, NH), 7.91 (s, 1H, CH), 7.83 (d, *J* = 8.3 Hz, 2H, 2 × C-H_arom_), 7.60 (d, *J* = 7.8 Hz, 1H, C-H_arom_), 7.45–7.42 (m, 3H, 3 × C-H_arom_), 7.20 (t, *J* = 8.1 Hz, 1H, C-H_arom_), 7.06 (t, *J* = 7.5 Hz, 1H, C-H_arom_), 6.80 (s, 1H, C_3_-H, indole), 3.02–2.91 (m, 1H, CH), 1.24 (d, *J* = 6.9 Hz, 6H, 2 × CH_3_); ^13^C NMR (100 MHz, DMSO-*d_6_*) δ (ppm): 151.8, 138.6, 138.2, 133.9, 131.7, 129.4 (two overlapping signals), 128.3, 127.7 (two overlapping signals), 123.8, 121.2, 120.4, 117.7, 111.8, 103.8, 102.7, 33.9, 24.0 (two overlapping signals); MS (ESI) *m*/*z*: 285 [M − H]^−^. Anal. calcd for C_20_H_18_N_2_ (286.37) (%): C, 83.88; H, 6.34; N, 9.78. Found: C, 83.96; H, 6.29; N, 9.75.
*2-(1H-Indol-2-yl)-3-(4-methoxyphenyl)acrylonitrile* (**2d**). Yield: 67% (yellow solid); m.p. 210–212 °C; IR ν_max_ (KBr, cm^−1^): 3352, 3045, 3009, 2949, 2927, 2831, 2222,1609, 1589, 1506, 1261, 1181, 1035, 814, 779, 729, 606; ^1^H NMR (400 MHz, DMSO-*d_6_*) δ (ppm): 11.67 (s, 1H, NH), 7.88 (d, *J* = 9.2 Hz, 3H, 2 × C-H_arom_ + CH), 7.58 (d, *J* = 7.8 Hz, 1H, C-H_arom_), 7.41 (d, *J* = 8.1 Hz, 1H, C-H_arom_), 7.23–7.13 (m, 3H, 3 × C-H_arom_), 7.04 (t, *J* = 7.5 Hz, 1H, C-H_arom_), 6.75 (s, 1H, C_3_-H, indole), 3.86 (s, 3H, CH_3_); ^13^C NMR (100 MHz, DMSO-*d_6_*) δ (ppm): 161.5, 138.6, 138.1, 134.1, 131.1 (two overlapping signals), 128.3, 126.6, 123.6, 121.1, 120.4, 118.0, 115.2 (two overlapping signals), 111.7, 103.2, 100.7, 55.9; MS (ESI) *m*/*z*: 273 [M − H]^−^. Anal. calcd for C_18_H_14_N_2_O (274.32): C, 78.81; H, 5.14; N, 10.21. Found: C, 78.75; H, 5.27; N, 10.19.
*3-(4-Ethoxyphenyl)-2-(1H-indol-2-yl)acrylonitrile* (**2e**). Yield: 58% (yellow solid); m.p. 174–175 °C; IR ν_max_ (KBr, cm^−1^): 3344, 3046, 2976, 2925, 2876. 2223, 1608, 1588, 1505, 1259, 1183, 1051, 779, 726; ^1^H NMR (400 MHz, DMSO-*d_6_*) δ (ppm): 11.67 (s, 1H, NH), 7.86–7.88 (m, 3H, C-H_arom_ + CH), 7.58 (d, *J* = 7.9 Hz, 1H, C-H_arom_), 7.41 (d, *J* = 8.9 Hz, 1H, C-H_arom_), 7.18 (t, *J* = 8.1 Hz, 1H, C-H_arom_), 7.11 (d, *J* = 8.8 Hz, 2H, 2 × C-H_arom_), 7.04 (t, *J* = 7.5 Hz, 1H, C-H_arom_), 6.74 (s, 1H, C_3_-H, indole), 4.12 (q, *J* = 7.0 Hz, 2H, CH_2_), 1.36 (t, *J* = 7.0 Hz, 3H, CH_3_); ^13^C NMR (100 MHz, DMSO-*d_6_*) δ (ppm): 160.8, 138.6, 138.1, 134.2, 131.2 (two overlapping signals), 128.4, 126.4, 123.5, 121.1, 120.3, 118.0, 115.6 (two overlapping signals), 111.7, 103.1, 100.6, 63.9, 15.0; MS (ESI) *m*/*z*: 287 [M − H]^−^. Anal. calcd for C_19_H_16_N_2_O (288.34): C, 79.14; H, 5.59; N, 9.72. Found: C, 78.95; H, 5.49; N, 9.85.
*3-(Benzo[d][1,3]dioxol-5-yl)-2-(1H-indol-2-yl)acrylonitrile* (**2f**). Yield: 15% (yellow solid); m.p. 233–234 °C; IR ν_max_ (KBr, cm^−1^): 3354, 3082, 3044, 2897, 2222, 1501, 1453, 1256, 1049, 782, 733, 622; ^1^H NMR (400 MHz, DMSO-*d_6_*) δ (ppm): 11.67 (s, 1H, NH), 7.83 (s, 1H, CH), 7.59 (d, *J* = 8.1 Hz, 1H, C-H_arom_), 7.55 (s, 1H, C-H_arom_), 7.41 (d, *J* = 8.1 Hz, 1H, C-H_arom_), 7.35 (d, *J* = 8.8 Hz, 1H, C-H_arom_), 7.19 (t, *J* = 7.0 Hz, 1H, C-H_arom_), 7.12 (d, *J* = 8.1 Hz, 1H, C-H_arom_), 7.05 (t, *J* = 7.0 Hz, 1H, C-H_arom_), 6.75 (s, 1H, C_3_-H, indole), 6.16 (s, 2H, CH_2_); ^13^C NMR (100 MHz, DMSO-*d_6_*) δ (ppm): 149.8, 148.5, 138.5, 138.2, 134.0, 128.3, 128.2, 126.0, 123.7, 121.1, 120.4, 117.9, 111.7, 109.5, 107.4, 103.4, 102.5, 101.2; MS (ESI) *m*/*z*: 287 [M − H]^−^. Anal. calcd for C_18_H_12_N_2_O_2_ (288.30) (%): C, 74.99; H, 4.20; N, 9.72. Found: C, 74.92; H, 4.32; N, 9.68.
*3-(4-Fluorophenyl)-2-(1H-indol-2-yl)acrylonitrile* (**2g**). Yield: 50% (yellow solid); m.p. 203–205 °C; IR ν_max_ (KBr, cm^−1^): 3349, 3062, 2224, 1603, 1593, 1504, 1242, 1164, 892, 818, 781, 729, 605; ^1^H NMR (400 MHz, DMSO-*d_6_*) δ (ppm): 11.73 (s, 1H, NH), 7.96–7.93 (m, 3H, 2 × C-H_arom_ + CH), 7.61 (d, *J* = 7.9 Hz, 1H, C-H_arom_), 7.44–7.40 (m, 3H, 3 × C-H_arom_), 7.21 (t, *J* = 7.1 Hz, 1H, C-H_arom_), 7.06 (t, *J* = 7.5 Hz, 1H, C-H_arom_), 6.82 (s, 1H, C_3_-H, indole); ^13^C NMR (100 MHz, DMSO-*d_6_*) δ (ppm): 163.39 (d, *J*_(C-F)_ = 250.0 Hz), 138.3, 137.5, 133.6, 131.59 (d, *J*_(C-F)_ = 8.0 Hz, two overlapping signals), 130.66 (d, *J*_(C-F)_ = 3.0 Hz), 128.2, 123.9, 121.3, 120.5, 117.5, 116.81 (d, *J*_(C-F)_ = 22.0 Hz, two overlapping signals), 111.8, 104.1, 103.6; MS (ESI) *m*/*z*: 261 [M − H]^−^; Anal. calcd for C_17_H_11_FN_2_ (262.28): C, 77.85; H, 4.23; N, 10.68. Found: C, 77.96; H, 4.25; N, 10.83.
*3-(2-Chlorophenyl)-2-(1H-indol-2-yl)acrylonitrile* (**2h**). Yield: 49% (yellow solid); m.p. 201–203 °C; IR ν_max_ (KBr, cm^−1^): 3355, 3059, 2225, 1593, 1433, 1340, 1304, 1258, 1045, 785, 747, 731, 611; ^1^H NMR (300 MHz, DMSO-*d_6_*) δ (ppm): 11.94 (s, 1H, NH), 8.05 (s, 1H, CH), 8.02–7.98 (m, 1H, C-H_arom_), 7.65–7.59 (m, 2H, 2 × C-H_arom_), 7.52–7.46 (m, 2H, 2 × C-H_arom_), 7.42 (d, *J* = 8.2 Hz, 1H, C-H_arom_), 7.21 (t, *J* = 7.1 Hz, 1H, C-H_arom_), 7.05 (t, *J* = 7.1 Hz, 1H, C-H_arom_), 6.86 (s, 1H, C_3_-H, indole); ^13^C NMR (75 MHz, DMSO-*d_6_*) δ (ppm): 138.5, 134.6, 133.8, 133.0, 132.6, 132.1, 130.3, 130.0, 128.1, 128.0, 124.4, 121.6, 120.6, 116.8, 111.9, 107.9, 105.4; MS (ESI) *m*/*z*: 277 [M − H]^−^. Anal. calcd for C_17_H_11_ClN_2_ (278.74) (%): C, 73.25; H, 3.98; Cl, 12.72; N, 10.05. Found: C, 73.29; H, 3.82; N, 10.15.
*3-(3-Chlorophenyl)-2-(1H-indol-2-yl)acrylonitrile* (**2i**). Yield: 22% (yellow solid); m.p. 188–189 °C; IR ν_max_ (KBr, cm^−1^): 3351, 3056, 2223, 1562, 1481, 1416, 1339, 1303, 1208, 1082, 784, 732, 674; ^1^H NMR (300 MHz, DMSO-*d_6_*) δ (ppm): 11.74 (s, 1H, NH), 7.89 (m, 2H, C-H_arom_ + CH), 7.82–7.77 (m, 1H, C-H_arom_), 7.60–7.53 (m, 3H, 3 × C-H_arom_), 7.42 (d, *J* = 8.1 Hz, 1H, C-H_arom_), 7.19 (t, *J* = 7.6 Hz, 1H, C-H_arom_), 7.04 (t, *J* = 8.0 Hz, 1H, C-H_arom_), 6.83 (s, 1H, C_3_-H, indole); ^13^C NMR (75 MHz, DMSO-*d_6_*) δ (ppm): 138.4, 136.7, 136.1, 134.2, 133.4, 131.5, 130.4, 128.4, 128.2, 127.9, 124.2, 121.5, 120.5, 117.1, 111.9, 105.3, 104.8; MS (ESI) *m*/*z*: 277 [M − H]^−^. Anal. calcd for C_17_H_11_ClN_2_ (278.74) (%): C, 73.25; H, 12.72; N, 10.05. Found: C, 73.17; H, 3.86; N, 10.12.
*3-(4-Chlorophenyl)-2-(1H-indol-2-yl)acrylonitrile* (**2j**). Yield: 67% (yellow solid); m.p. 241–242 °C; IR ν_max_ (KBr, cm^−1^): 3357, 3082, 3050, 3015, 2225, 1597, 1585, 1488, 1409, 1101, 806, 787, 732; ^1^H NMR (300 MHz, DMSO-*d_6_*) δ (ppm): 11.73 (s, 1H, NH), 7.89–7.85 (m, 3H, 2 × C-H_arom_ + CH), 7.62–7.57 (m, 3H, 3 × C-H_arom_), 7.40 (d, *J* = 8.0 Hz, 1H, C-H_arom_), 7.19 (t, *J* = 7.3 Hz, 1H, C-H_arom_), 7.04 (t, *J* = 7.2 Hz, 1H, C-H_arom_), 6.81 (s, 1H, C_3_-H, indole); ^13^C NMR (75 MHz, DMSO-*d_6_*) δ (ppm): 138.3, 137.1, 135.3, 133.5, 132.9, 130.8 (two overlapping signals), 129.7 (two overlapping signals), 128.2, 124.1, 121.4, 120.5, 117.3, 111.8, 104.5, 104.3; MS (ESI) *m*/*z*: 274 [M − H]^−^. Anal. calcd for C_17_H_11_ClN_2_ (278.74) (%): C, 73.25; H, 3.98; N, 10.05. Found: C, 73.43; H, 3.75; N, 9.85.
*3-(4-Bromophenyl)-2-(1H-indol-2-yl)acrylonitrile* (**2k**). Yield: 46% (yellow solid); m.p. 239–241 °C; IR ν_max_ (KBr, cm^−1^): 3358, 3082, 3056, 3019, 2225, 1581, 1485, 1406, 1078, 1010, 894, 803, 782, 733, 608; ^1^H NMR (400 MHz, DMSO-*d_6_*) δ (ppm): 11.76 (s, 1H, NH), 7.90 (s, 1H, CH), 7.83–7.76 (m, 4H, 4 × C-H_arom_), 7.61 (d, *J* = 7.9 Hz, 1H, C-H_arom_), 7.43 (d, *J* = 9.0 Hz, 1H, C-H_arom_), 7.22 (t, *J* = 7.6 Hz, 1H, C-H_arom_), 7.06 (t, *J* = 8.0 Hz, 1H, C-H_arom_), 6.84 (s, 1H, C_3_-H, indole); ^13^C NMR (100 MHz, DMSO-*d_6_*) δ (ppm): 138.3, 137.2, 133.6, 133.2, 132.7 (two overlapping signals), 131.0 (two overlapping signals), 128.2, 124.2, 124.1, 121.4, 120.5, 117.3, 111.9, 104.6, 104.4; MS (ESI) *m*/*z*: 322 [M − H]^−^; Anal. calcd for C_17_H_11_BrN_2_ (323.19): C, 63.18; H, 3.43; N, 8.67. Found: C, 62.97; H, 3.59; N, 8.53.
*3-[4-(Dimethylamino)phenyl]-2-(1H-indol-2-yl)acrylonitrile* (**2l**). Yield: 37% (brown solid); m.p. 234–236 °C; IR ν_max_ (KBr, cm^−1^): 3334, 3079, 3013, 2989, 2814, 2211, 1611, 1575, 1364, 1198, 810, 788, 746; ^1^H NMR (400 MHz, DMSO-*d_6_*) δ (ppm): 11.55 (s, 1H, NH), 7.80 (d, *J* = 8.9 Hz, 2H, 2 × C-H_arom_), 7.75 (s, 1H, CH), 7.55 (d, *J* = 7.8 Hz, 1H, C-H_arom_), 7.39 (d, *J* = 8.1 Hz, 1H, C-H_arom_), 7.15 (t, *J* = 7.5 Hz, 1H, C-H_arom_), 7.02 (t, *J* = 7.4 Hz, 1H, C-H_arom_), 6.84 (d, *J* = 9.0 Hz, 2H, 2 × C-H_arom_), 6.64 (s, 1H, C_3_-H, indole), 3.03 (s, 6H, 2 × CH_3_); ^13^C NMR (100 MHz, DMSO-*d_6_*) δ (ppm): 152.1, 139.4, 138.0, 135.0, 131.1 (two overlapping signals), 128.6, 123.0, 121.2, 120.7, 120.2, 118.9, 112.3 (two overlapping signals), 111.5, 101.7, 96.3, 40.1 (two overlapping signals); MS (ESI) *m*/*z*: 286 [M − H]^−^. Anal. calcd for C_19_H_17_N_3_ (287.36) (%): C, 79.41; H, 5.96; N, 14.62. Found: C, 79.31; H, 5.87; N, 14.82.
Crystal data for **2l**: C_19_H_17_N_3_ (*M* = 287.35 g/mol), monoclinic, space group P2_1_/n, *a* = 6.76580(10) Å, *b* = 21.9867(4) Å, *c* = 10.2650(2) Å, *β* = 93.006(2)°, *V* = 1524.90(5) Å^3^, *Z* = 4, μ(Cu Kα) = 0.587 mm^−1^, *D_calc_* = 1.252 g/cm^3^, 6428 reflections measured, 3114 unique (*R*_int_ = 0.0141, R_sigma_ = 0.0179), which were used in all calculations. The final *R*_1_ was 0.0412 (I > 2σ(I)) and *wR*_2_ was 0.1225 (all data).
*3-[4-(Diethylamino)phenyl]-2-(1H-indol-2-yl)acrylonitrile* (**2m**). Yield: 7% (brown solid); m.p. 183–185 °C; IR ν_max_ (KBr, cm^−1^): 3336, 3047, 2967, 2925, 2866, 2219, 1609, 1584, 1512, 1356, 1200, 812, 799, 731; ^1^H NMR (400 MHz, DMSO-*d_6_*) δ (ppm): 11.54 (s, 1H, NH), 7.78 (d, *J* = 8.9 Hz, 2H, 2 × C-H_arom_), 7.72 (s, 1H, CH), 7.54 (d, *J* = 7.8 Hz, 1H, C-H_arom_), 7.38 (d, *J* = 8.1 Hz, 1H, C-H_arom_), 7.14 (t, *J* = 7.4 Hz, 1H, C-H_arom_), 7.02 (t, *J* = 7.4 Hz, 1H, C-H_arom_), 6.81 (d, *J* = 8.9 Hz, 2H, 2 × C-H_arom_), 6.62 (s, 1H, C_3_-H, indole), 3.44 (q, *J* = 6.9 Hz, 4H, 2 × CH_2_), 1.14 (t, *J* = 6.9 Hz, 6H, 2 × CH_3_); ^13^C NMR (100 MHz, DMSO-*d_6_*) δ (ppm): 149.6, 139.4, 137.9, 135.1, 131.5 (two overlapping signals), 128.6, 122.9, 120.7, 120.4, 120.2, 119.0, 111.8 (two overlapping signals), 111.5, 101.5, 95.5, 44.3 (two overlapping signals), 12.9 (two overlapping signals); MS (ESI) *m*/*z*: 314 [M − H]^−^; MS (ESI) *m*/*z*: 316 [M + H]^+^. Anal. calcd for C_21_H_21_N_3_ (315.41) (%): C, 79.97; H, 6.71; N, 13.32. Found: C, 79.75; H, 6.87; N, 13.38.
*2-(1H-Indol-2-yl)-3-(4-nitrophenyl)acrylonitrile* (**2n**). Yield: 27% (orange solid); m.p. 210–212 °C; IR ν_max_ (KBr, cm^−1^): 3344, 2227, 1595, 1578, 1512, 1341, 1111, 787, 753, 684; ^1^H NMR (300 MHz, DMSO-*d_6_*) δ (ppm): 11.83 (s, 1H, NH), 8.36 (d, *J* = 8.9 Hz, 2H, C-H_arom_), 8.05 (d, *J* = 8.9 Hz, 2H, 2 × C-H_arom_), 8.00 (s, 1H, CH), 7.61 (d, *J* = 7.9 Hz, 1H, C-H_arom_), 7.42 (d, *J* = 7.7 Hz, 1H, C-H_arom_), 7.22 (t, *J* = 7.6 Hz, 1H, C-H_arom_), 7.05 (t, *J* = 7.9 Hz, 1H, C-H_arom_), 6.91 (s, 1H, C3-H, indole); ^13^C NMR (75 MHz, DMSO-*d_6_*) δ (ppm): 148.0, 140.2, 138.6, 135.6, 133.3, 130.2 (two overlapping signals), 128.1, 124.7, 124.6, 121.7, 120.7, 115.8, 114.9, 112.0, 107.2, 105.9; MS (ESI) *m*/*z*: 288 [M − H]^−^. Anal. calcd for C_17_H_11_N_3_O_2_ (289.29) (%): C, 70.58; H, 3.83; N, 14.53. Found: C, 70.67; H, 3.65; N, 14.87.
*2-(1H-Indol-2-yl)-3-(naphthalen-1-yl)acrylonitrile* (**2o**). Yield: 46% (yellow solid); m.p. 218–220 °C; IR ν_max_ (KBr, cm^−1^): 3372, 3058, 3013, 2921, 2847, 2222, 1587, 1431, 1301, 882, 777, 728, 674; ^1^H NMR (400 MHz, DMSO-*d_6_*) δ (ppm): 11.98 (s, 1H), 8.67 (s, 1H, CH), 8.31 (d, *J* = 8.2 Hz, 1H, C-H_arom_), 8.13–8.05 (m, 3H, 3 × C-H_arom_), 7.71–7.64 (m, 4H, 4 × C-H_arom_), 7.48 (d, *J* = 8.2 Hz, 1H, C-H_arom_), 7.25 (t, *J* = 7.6 Hz, 1H, C-H_arom_), 7.09 (t, *J* = 7.5 Hz, 1H, C-H_arom_), 6.89 (s, 1H, C_3_-H, indole); ^13^C NMR (100 MHz, DMSO-*d_6_*) δ (ppm): 138.4, 136.4, 133.7, 133.7, 131.6, 131.2, 131.0, 129.3, 128.2, 127.6, 127.2, 126.9, 126.1, 124.3, 124.2, 121.5, 120.5, 117.4, 111.8, 107.1, 104.8; MS (ESI) *m*/*z*: 293 [M − H]^−^. Anal. calcd for C_21_H_14_N_2_ (294.35) (%): C, 85.69; H, 4.79; N, 9.52. Found: C, 85.58; H, 4.82; N, 9.32.
*2-(1H-Indol-2-yl)-3-(naphthalen-2-yl)acrylonitrile* (**2p**). Yield: 50% (yellow solid); m.p. 229–230 °C; IR ν_max_ (KBr, cm^−1^): 3364, 3052, 2920, 2224, 1594, 1424, 1342, 915, 818, 769, 734, 673; ^1^H NMR (400 MHz, DMSO-*d_6_*) δ (ppm): 11.83 (br. s, 1H, NH), 8.35 (s, 1H, CH), 8.11–8.09 (m, 3H, 3 × C-H_arom_), 8.06–8.03 (m, 1H, C-H_arom_), 8.01–7.98 (m, 1H, C-H_arom_), 7.66–7.60 (m, 3H, 3 × C-H_arom_), 7.47–7.44 (m, 1H, C-H_arom_), 7.22 (t, *J* = 7,1 Hz, 1H, C-H_arom_), 7.07 (t, *J* = 7.1 Hz, 1H, C-H_arom_), 6.87 (s, 1H, C_3_-H, indole); ^13^C NMR (100 MHz, DMSO-*d_6_*) δ (ppm): 138.5, 138.4, 133.9, 133.2, 131.7, 130.2, 129.2, 129.1, 128.3, 128.24, 128.20, 127.6, 125.1, 124.0, 121.4, 120.5, 117.7, 111.9, 104.3, 103.9; MS (ESI) *m*/*z*: 293 [M − H]^−^. Anal. calcd for C_21_H_14_N_2_ (294.35) (%): C, 85.69; H, 4.79; N, 9.52. Found: C, 85.75; H, 4,67; N, 9.58.
*2-(1H-Indol-2-yl)-3-(pyridin-2-yl)acrylonitrile* (**2q**). Yield: 24% (yellow solid); m.p. 179–181 °C; IR ν_max_ (KBr, cm^−1^): 3318, 3056, 3008, 2924, 2229, 1577, 1423, 1305, 1149, 895, 787, 723, 608; ^1^H NMR (300 MHz, DMSO-*d_6_*) δ (ppm): 11.81 (s, 1H, NH), 8.73 (m, 1H, C-H_arom_), 7.98–7.90 (m, 2H, C-H_arom_ + CH), 7.67 (d, *J* = 7.9 Hz, 1H, C-H_arom_), 7.60 (d, *J* = 7.9 Hz, 1H, C-H_arom_), 7.45–7.40 (m, 2H, 2 × C-H_arom_), 7.20 (t, *J* = 7.6 Hz, 1H, C-H_arom_), 7.04 (t, *J* = 7.5 Hz, 1H, C-H_arom_), 6.90 (s, 1H, C_3_-H, indole); ^13^C NMR (75 MHz, DMSO-*d_6_*) δ (ppm): 152.0, 150.3, 138.5, 137.8, 136.4, 133.9, 128.2, 125.7, 125.0, 124.3, 121.5, 120.5, 117.0, 111.9, 106.3, 105.5; MS (ESI) *m*/*z*: 244 [M − H]^−^; MS (ESI) *m*/*z*: 246 [M + H]^+^. Anal. calcd for C_16_H_11_N_3_ (245.28) (%): C, 78.35; H, 4.52; N, 17.13. Found: C, 78.27; H, 4.38; N, 17.35.
*2-(1H-Indol-2-yl)-3-(quinolin-2-yl)acrylonitrile* (**2r**). Yield: 17% (orange solid); m.p. > 300 °C; IR ν_max_ (KBr, cm^−1^): 3329, 3048, 2923, 2853, 2225, 1587, 1548, 1343, 886, 829, 796, 751, 728; ^1^H NMR (400 MHz, DMSO-*d_6_*) δ (ppm): 11.93 (s, 1H, NH), 8.53 (d, *J* = 8.4 Hz, 1H, C-H_arom_), 8.11 (s, 1H, CH), 8.06 (t, *J* = 8.6 Hz, 2H, 2 × C-H_arom_), 7.89–7.84 (m, 2H, 2 × C-H_arom_), 7.71–7.65 (m, 2H, 2 × C-H_arom_), 7.46 (d, *J* = 8.2 Hz, 1H, C-H_arom_), 7.25 (t, *J* = 7.1 Hz, 1H, C-H_arom_), 7.09 (t, *J* = 7.0 Hz, 1H, C-H_arom_), 7.00 (s, 1H, C_3_-H, indole); MS (ESI) *m*/*z*: 294 [M − H]^−^; MS (ESI) *m*/*z*: 296 [M + H]^+^. Anal. calcd for C_20_H_13_N_3_ (295.34) (%): C, 81.34; H, 4.44; N, 14.23. Found: C, 81.42; H, 4.32; N, 14.26.
*2-(1H-Indol-2-yl)-3-(thiophen-2-yl)acrylonitrile* (**2s**). Yield: 21% (yellow solid); m.p. 178–180 °C; IR ν_max_ (KBr, cm^−1^): 3351 3107, 3040, 2924, 2853, 2220, 1577, 1225, 1408, 1301, 885, 779, 691, 607; ^1^H NMR (400 MHz, DMSO-*d_6_*) δ (ppm): 11.70 (s, 1H, NH), 8.13 (s, 1H, CH), 7.92 (d, *J* = 5.6 Hz, 1H, C-H_arom_), 7.68 (d, *J* = 4.6 Hz, 1H, C-H_arom_), 7.59 (d, *J* = 8.1 Hz, 1H, C-H_arom_), 7.41 (d, *J* = 9.0 Hz, 1H, C-H_arom_), 7.28–7.30 (m, 1H, C-H_arom_), 7.19 (t, *J* = 8.1 Hz, 1H, C-H_arom_), 7.05 (t, *J* = 7.1 Hz, 1H, C-H_arom_) 6.76 (s, 1H, C_3_-H, indole); ^13^C NMR (100 MHz, DMSO-*d_6_*) δ (ppm): 138.3, 137.8, 133.6, 133.4, 131.8, 131.7, 128.8, 128.4, 123.8, 121.2, 120.5, 117.7, 111.7, 103.8, 100.1; MS (ESI) *m*/*z*: 249 [M − H]^−^. Anal. calcd for C_15_H_10_N_2_S (250.32) (%): C, 71.97; H, 4.03; N, 11.19. Found: C, 71.85; H, 3.97; N, 11.27.

#### 3.1.3. General Procedure for the Preparation of 2-(1H-Indol-2-yl)-3-acrylonitriles **2t**–**x**

To a stirred solution of 2-(1*H*-indol-2-yl)acetonitrile (**1**) (312 mg, 2.0 mmol) in anhydrous ethanol (2 mL) was added the appropriate carboxaldehyde (3.0 mmol) and ten drops of triethylamine. Stirring was continued under reflux for 15 min and then at ambient temperature for 48 h. The precipitate thus obtained was collected by vacuum filtration, washed with anhydrous ethanol, and dried. In this manner, the following compounds were obtained.

*3-(5-Chlorothiophen-2-yl)-2-(1H-indol-2-yl)acrylonitrile* (**2t**). Yield: 33% (green solid); m.p. 259–261 °C; IR ν_max_ (KBr, cm^−1^): 3348, 3054, 2218, 1501, 1433, 1414, 1308, 1228, 786, 741, 608; ^1^H NMR (400 MHz, DMSO-*d_6_*) δ (ppm): 11.70 (s, 1H, NH), 8.03 (s, 1H, CH), 7.59 (d, *J* = 7.9 Hz, 1H, C-H_arom_), 7.51–7.49 (m, 1H, CH_arom_), 7.40 (d, *J* = 8.1 Hz, 1H, C-H_arom_), 7.34–7.32 (m, 1H, C-H_arom_), 7.19 (t, *J* = 7.6 Hz, 1H, C-H_arom_), 7.05 (t, *J* = 7.4 Hz, 1H), 6.77 (s, 1H, C_3_-H, indole); MS (ESI) *m*/*z*: 155 [M − H]^−^. Anal. calcd for C_15_H_9_ClN_2_S (284.76) (%): C, 63.27; H, 3.19; N, 9.84. Found: C, 63.15; H, 3.21; N, 9.72.
*3-(Furan-2-yl)-2-(1H-indol-2-yl)acrylonitrile* (**2u**). Yield: 53% (yellow solid); m.p. 185–186 °C; IR ν_max_ (KBr, cm^−1^): 3321, 3056, 2924, 2231, 1607, 1469, 1346, 1303, 1019, 884, 781, 724; ^1^H NMR (400 MHz, DMSO-*d_6_*) δ (ppm): 11.71 (s, 1H, NH), 8.02 (s, 1H, CH), 7.76 (s, 1H, C-H_arom_), 7.58 (d, *J* = 7.9 Hz, 1H, C-H_arom_), 7.39 (d, *J* = 8.0 Hz, 1H, C-H_arom_), 7.19 (t, *J* = 7.6 Hz, 1H, C-H_arom_), 7.15 (d, *J* = 3.5 Hz, 1H, C-H_arom_), 7.05 (t, *J* = 7.3 Hz, 1H, C-H_arom_), 6.82–6.74 (m, 2H, 2 × C-H_arom_); ^13^C NMR (100 MHz, DMSO-*d_6_*) δ (ppm): 149.9, 146.7, 138.3, 133.5, 128.3, 124.7, 123.9, 121.2, 120.5, 117.3, 116.4, 113.8, 111.7, 104.0, 99.6; MS (ESI) *m*/*z*: 233 [M − H]^−^. Anal. calcd for C_15_H_10_N_2_O (234.25) (%): C, 76.91; H, 4.30; N, 11.96. Found: C, 76.83; H, 4.38; N, 11.86.
*2-(1H-Indol-2-yl)-3-(5-methylfuran-2-yl)acrylonitrile* (**2v**). Yield: 45% (yellow solid); m.p. 190–191 °C; IR ν_max_ (KBr, cm^−1^): 3322, 3053, 2920, 2850, 2226, 1517, 1539, 1489, 1290, 1025, 781, 748; ^1^H NMR (400 MHz, DMSO-*d_6_*) δ (ppm): 11.64 (s, 1H, NH), 7.67 (s, 1H, CH), 7.56 (d, *J* = 7.9 Hz, 1H, C-H_arom_), 7.38 (d, *J* = 8.2 Hz, 1H, C-H_arom_), 7.17 (t, *J* = 7.6 Hz, 1H, C-H_arom_), 7.08–7.01 (m, 2H, 2 × C-H_arom_), 6.73 (s, 1H, C_3_-H, indole), 6.43 (d, *J* = 3.2 Hz, 1H, C-H_arom_), 2.41 (s, 3H, CH_3_); MS (ESI) *m*/*z*: 247 [M − H]^−^. Anal. calcd for C_16_H_12_N_2_O (248.28) (%): C, 77.40; H, 4.87; N, 11.28. Found: C, 77.46; H, 4.75; N, 11.32.
*3-(5-Chlorofuran-2-yl)-2-(1H-indol-2-yl)acrylonitrile* (**2w**). Yield: 26% (yellow solid); m.p. 223–228 °C; IR ν_max_ (KBr, cm^−1^): 3333, 3045, 2227, 1519, 1469, 1345, 1020, 876, 774, 729; ^1^H NMR (400 MHz, DMSO-*d_6_*) δ (ppm): 11.71 (s, 1H, NH), 7.67 (s, 1H, CH), 7.59 (d, *J* = 7.9 Hz, 1H, C-H_arom_), 7.39 (d, *J* = 8.1 Hz, 1H, C-H_arom_), 7.23–7.16 (m, 2H, 2 × C-H_arom_), 7.05 (t, *J* = 7.8 Hz, 1H, C-H_arom_), 6.82 (d, *J* = 3.6 Hz, 1H, C-H_arom_), 6.80 (s, 1H, C_3_-H, indole); MS (ESI) *m*/*z*: 267 [M − H]^−^. Anal. calcd. for C_15_H_9_ClN_2_O (268.70) (%): C, 67.05; H, 3.38; N, 10.43. Found: C, 67.17; H, 3.46; N, 10.37.
*2-(1H-Indol-2-yl)-3-(1H-pyrrol-2-yl)acrylonitrile* (**2x**). Yield: 8% (brown, solid); m.p. 134–136 °C; IR ν_max_ (KBr, cm^−1^): 3427, 3331, 3113, 3014, 2924, 2215, 1596, 1402, 1342, 1038, 786, 748, 663, 544; ^1^H NMR (400 MHz, DMSO-*d_6_*) δ (ppm): 11.62 (s, 1H, NH), 11.54 (s, 1H, NH), 7.64 (s, 1H, CH), 7.54 (d, *J* = 7.7 Hz, 1H, C-H_arom_), 7.38 (d, *J* = 8.0 Hz, 1H, C-H_arom_), 7.16–7.12 (m, 3H, 3 × C-H_arom_), 7.02 (t, *J* = 7.3 Hz, 1H, C-H_arom_), 6.62 (s, 1H, C_3_-H, indole), 6.36 (s, 1H, C-H_arom_); ^13^C NMR (100 MHz, DMSO-*d_6_*) δ (ppm): 138.0, 134.4, 129.4, 128.6, 127.6, 124.2, 122.9, 120.6, 120.2, 118.8, 112.9, 111.7, 111.6, 101.6, 95.2; MS (ESI) *m*/*z*: 232 [M − H]^−^; MS (ESI) *m*/*z*: 234 [M + H]^+^. Anal. calcd for C_15_H_11_N_3_ (233.27) (%): C, 77.23; H, 4.75; N, 18.01. Found: C, 77.31; H, 4.83; N, 17.86.

#### 3.1.4. Synthesis of N-[4-(Dimethylamino)phenyl]-1H-indole-2-carbimidoyl cyanide (**3**)

To a solution of 2-(1*H*-indol-2-yl)acetonitrile (**1**) (312 mg, 2.0 mmol) in anhydrous methanol (10 mL) was added dropwise a solution of sodium methoxide (60 mg of sodium in 6 mL of anhydrous methanol) and the reaction mixture was stirred at ambient temperature for 30 min. Then, 4-nitroso-*N*,*N*-dimethylaniline (600 mg, 4.0 mmol) was added and stirring was continued at ambient temperature for 5 h. The product **3** that precipitated was filtered, washed with methanol, and dried. Yield: 42% (brown solid); m.p. 181–183 °C; IR νmax (KBr, cm^−1^): 3399, 3049, 2888, 2805, 2220, 1608, 1510, 1361, 1339, 1220, 1177, 824, 785, 743, 688; ^1^H NMR (400 MHz, DMSO-*d_6_*) δ (ppm): 11.91 (s, 1H, NH), 7.69 (d, *J* = 7.9 Hz, 1H, C-H_arom_), 7.48–7.46 (m, 3H, 3 × C-H_arom_), 7.27 (t, *J* = 7.6 Hz, 1H, C-H_arom_), 7.14 (s, 1H, C3-H, indole), 7.09 (t, *J* = 7.4 Hz, 1H, C-H_arom_), 6.85 (d, *J* = 9.1 Hz, 2H, 2 × C-H_arom_), 3.02 (s, 6H, 2 × CH_3_); ^13^C NMR (100 MHz, DMSO-*d_6_*) δ (ppm): 150.8, 138.9, 136.9, 135.4, 127.9, 125.4, 124.5 (two overlapping signals), 124.3, 122.3, 120.7, 113.2, 112.7, 112.5 (two overlapping signals), 108.6, 40.4 (two overlapping signals); MS (ESI) m/z: 287 [M − H]^−^. Anal. calcd for C_18_H_16_N_4_ (288.35) (%): C, 74.98; H, 5.59; N, 19.43. Found: C, 75.02; H, 5.51; N, 19.47.

#### 3.1.5. General Procedure for the Preparation of 2-(1H-Indol-2-yl)-3-phenylpropanenitriles **4a**–**c**

To a suspension of sodium borohydride (57 mg, 1.5 mmol) in a mixture of dimethylformamide (4 mL) and methanol (1 mL) was added the appropriate 2-(1*H*-indol-2-yl)-3-acrylonitrile **2d**, **2l**, or **2p** (0.75 mmol). The mixture was stirred overnight and then diluted with water, neutralized with hydrochloric acid, and extracted with dichloromethane. The organic phase was dried (Na_2_SO_4_), concentrated under vacuum, and subjected to preparative thin-layer chromatography eluting with petroleum ether/ethyl acetate (9:2 *v*/*v*). In this manner, the following compounds were obtained.

*2-(1H-Indol-2-yl)-3-(4-methoxyphenyl)propanenitrile* (**4a**). Yield: 73% (white solid); m.p. 151–153 °C; IR ν_max_ (KBr, cm^−1^): 3397, 3044, 3009, 2935, 2839, 2242, 1612, 1512, 1242, 1177, 1032, 800, 739, 667; ^1^H NMR (400 MHz, DMSO-*d_6_*) δ (ppm): 11.43 (s, 1H, NH), 7.50 (d, *J* = 7.9 Hz, 1H, C-H_arom_), 7.39 (d, *J* = 8.9 Hz, 1H, C-H_arom_), 7.21 (d, *J* = 8.7 Hz, 2H, 2 × C-H_arom_), 7.11 (t, *J* = 7.0 Hz, 1H, C-H_arom_), 7.00 (t, *J* = 7.5 Hz, 1H, C-H_arom_), 6.88 (d, *J* = 8.7 Hz, 2H, 2 × C-H_arom_), 6.39 (s, 1H, C_3_-H, indole), 4.72–4.65 (m, 1H, CH), 3.73 (s, 3H, CH_3_), 3.32–3.18 (m, 2H, CH_2_); ^13^C NMR (100 MHz, DMSO-*d_6_*) δ (ppm): 158.7, 136.9, 133.2, 130.6 (two overlapping signals), 129.3, 127.8, 122.0, 120.5, 120.4, 119.7, 114.2 (two overlapping signals), 111.7, 100.8, 55.4, 37.7, 33.0; MS (ESI) *m*/*z*: 275 [M − H]^−^. Anal. calcd for C_18_H_16_N_2_O (276.33): C, 78.24; H, 5.84; N, 10.14; O, 5.79. Found: C, 78.32; H, 5.92; N, 10.06.
*3-[4-(Dimethylamino)phenyl]-2-(1H-indol-2-yl)propanenitrile* (**4b**). Yield: 75% (beige solid); m.p. 180–183 °C; IR ν_max_ (KBr, cm^−1^): 3400, 3355, 3048, 2923, 2857, 2810, 2240, 1613, 1524, 1357, 1344, 1191, 796, 752, 743, 667; ^1^H NMR (400 MHz, DMSO-*d_6_*) δ (ppm): 11.42 (s, 1H, NH), 7.50 (d, *J* = 7.9 Hz, 1H, C-H_arom_), 7.40 (d, *J* = 8.1 Hz, 1H, C-H_arom_), 7.14–7.09 (m, 3H, 3 × C-H_arom_), 7.00 (t, *J* = 7.0 Hz, 1H, C-H_arom_), 6.67 (d, *J* = 8.7 Hz, 2H, 2 × C-H_arom_), 6.40 (s, 1H, C_3_-H, indole), 4.60–4.64 (m, 1H, CH), 3.28–3.14 (m, 2H, CH_2_), 2.86 (s, 6H, 2 × CH_3_); MS (ESI) *m*/*z*: 288 [M − H]^−^. Anal. calcd for C_19_H_19_N_3_ (289.37) (%): C, 78.86; H, 6.62; N, 14.52. Found: C, 78.76; H, 6.58; N, 14.66.
*2-(1H-Indol-2-yl)-3-(naphthalen-2-yl)propanenitrile* (**4c**). Yield: 63% (beige solid); m.p. 164–166 °C; IR ν_max_ (KBr, cm^−1^): 3392, 3046, 2924, 2242, 1455, 1426, 1290, 802, 752, 743, 667; ^1^H NMR (300 MHz, DMSO-*d_6_*) δ (ppm): 11.50 (s, 1H, NH), 7.88–7.83 (m, 4H, 4 × C-H_arom_), 7.51–7.38 (m, 5H, 5 × C-H_arom_), 7.10 (t, *J* = 7.7 Hz, 1H, C-H_arom_), 6.98 (t, *J* = 7.6 Hz, 1H, C-H_arom_), 6.42 (s, 1H, C_3_-H, indole), 4.90–4.85 (m, 1H, CH), 3.58–3.41 (m, 2H, CH_2_); ^13^C NMR (75 MHz, DMSO-*d_6_*) δ (ppm): 136.9, 135.1, 133.3, 133.1, 132.5, 128.4, 128.1, 128.0, 127.9, 127.8, 127.7, 126.7, 126.3, 122.0, 120.5, 120.3, 119.7, 111.7, 100.9, 38.6, 32.6; MS (ESI) *m*/*z*: 295 [M − H]^−^. Anal. calcd for C_21_H_16_N_2_ (296.37) (%): C, 85.11; H, 5.44; N, 9.45. Found: C, 85.23; H, 5.38; N, 9.39.

#### 3.1.6. General Procedure for the Preparation of 2-(1-Methyl-1*H*-indol-2-yl)-3-acrylonitriles **5a**–**d**

To a stirred solution of the appropriate 2-(1*H*-indol-2-yl)-3-acrylonitrile derivative **2b**, **2d**, **2l**, or **2p** (0.75 mmol) in anhydrous dimethylformamide (5 mL) was added sodium hydride (35 mg, 0.9 mmol, 60% oil dispersion) in one portion at 0 °C. The reaction mixture was stirred for an additional 30 min at ambient temperature. Then, the reaction mixture was cooled to 0 °C and treated with methyl iodide (124 mg, 54 µL, 0.9 mmol). After stirring overnight at ambient temperature, the mixture was diluted with water and the resulting precipitate was collected by filtration, washed with water, and dried. In this manner, the following compounds were obtained.

*2-(1-Methyl-1H-indol-2-yl)-3-(p-tolyl)acrylonitrile* (**5a**). Yield: 77% (beige solid); m.p. 90–92 °C; IR ν_max_ (KBr, cm^−1^): 3040, 2914, 2854, 2223, 1468, 1359, 1343, 1187, 815, 785, 750, 731; ^1^H NMR (400 MHz, DMSO-*d_6_*) δ (ppm): 7.89 (d, *J* = 8.2 Hz, 2H, 2 × C-H_arom_), 7.73 (s, 1H, CH), 7.61 (d, *J* = 7.9 Hz, 1H, C-H_arom_), 7.54 (d, *J* = 8.3 Hz, 1H, C-H_arom_), 7.38 (d, *J* = 8.1 Hz, 2H, 2 × C-H_arom_), 7.26 (t, *J* = 7.3 Hz, 1H, C-H_arom_), 7.11 (t, *J* = 7.3 Hz, 1H, C-H_arom_), 6.80 (s, 1H, C_3_-H, indole), 3.87 (s, 3H, CH_3_), 2.40 (s, 3H, CH_3_); ^13^C NMR (100 MHz, DMSO-*d_6_*) δ (ppm): 146.9, 141.7, 139.1, 135.4, 131.2, 130.1 (two overlapping signals), 129.7 (two overlapping signals), 127.2, 123.3, 121.1, 120.6, 118.2, 110.8, 104.0, 101.0, 31.7, 21.6. Anal. calcd for C_19_H_16_N_2_ (272.34) (%): C, 83.79; H, 5.92; N, 10.29. Found: C, 83.73; H, 5.86; N, 10.41.
*3-(4-Methoxyphenyl)-2-(1-methyl-1H-indol-2-yl)acrylonitrile* (**5b**). Yield: 96% (yellow solid); m.p. 113–114 °C; IR ν_max_ (KBr, cm^−1^): 3050, 3000, 2924, 2853, 2210, 1601, 1506, 1461, 1253, 1180, 1031, 828, 798, 754, 744; ^1^H NMR (400 MHz, DMSO-*d_6_*) δ (ppm): 7.98 (d, *J* = 8.8 Hz, 2H, 2 × C-H_arom_), 7.69 (s, 1H, CH), 7.60 (d, *J* = 7.8 Hz, 1H, C-H_arom_), 7.52 (d, *J* = 8.3 Hz, 1H, C-H_arom_), 7.25 (t, *J* = 7.1 Hz, 1H, C-H_arom_), 7.15–7.11 (m, 3H, 3 × C-H_arom_), 6.76 (s, 1H, C_3_-H, indole), 3.86 (s, 6H, NCH_3_ + OCH_3_); ^13^C NMR (100 MHz, DMSO-*d_6_*) δ (ppm): 161.9, 146.8, 139.0, 135.6, 131.7 (two overlapping signals), 127.3, 126.5, 123.1, 121.0, 120.6, 118.5, 115.1 (two overlapping signals), 110.8, 103.6, 98.9, 56.0, 31.7. Anal. calcd for C_19_H_16_N_2_O (288.34) (%): C, 79.14; H, 5.59; N, 9.72. Found: C, 79.22; H, 5.51; N; 9.68.
*3-[4-(Dimethylamino)phenyl]-2-(1-methyl-1H-indol-2-yl)acrylonitrile* (**5c**). Yield: 92% (brown solid); m.p. 131–132 °C; IR ν_max_ (KBr, cm^−1^): 3106, 3035, 2887, 2804, 2218, 1608, 1588, 1361, 1197, 818, 792, 732; ^1^H NMR (400 MHz, DMSO-*d_6_*) δ (ppm): 7.89 (d, *J* = 8.9 Hz, 2H, 2 × C-H_arom_), 7.57 (d, *J* = 7.8 Hz, 1H, C-H_arom_), 7.52–7.49 (m, 2H, C-H_arom_ + CH), 7.22 (t, *J* = 7.3 Hz, 1H, C-H_arom_), 7.09 (t, *J* = 7.4 Hz, 1H, C-H_arom_), 6.83 (d, *J* = 9.0 Hz, 2H, 2 × C-H_arom_), 6.67 (s, 1H, C_3_-H, indole), 3.84 (s, 3H, CH_3_), 3.04 (s, 6H, 2 × CH_3_); ^13^C NMR (100 MHz, DMSO-*d_6_*) δ (ppm): 152.4, 147.6, 138.7, 136.5, 131.7 (two overlapping signals), 127.4, 122.6, 121.1, 120.7, 120.4, 119.4, 112.1 (two overlapping signals), 110.6, 102.7, 93.9, 40.1 (two overlapping signals), 31.6; MS (ESI) *m*/*z*: 302 [M + H]^+^. Anal. calcd. for C_20_H_19_N_3_ (301.38) (%): C, 79.70; H, 6.35; N, 13.94. Found: C, 79.76; H, 6.27; N, 13.97.
*2-(1-Methyl-1H-indol-2-yl)-3-(naphthalen-2-yl)acrylonitrile* (**5d**). Yield: 84% (yellow solid); m.p. 160–163 °C; IR ν_max_ (KBr, cm^−1^): 3054, 2925, 2853, 2207, 1599, 1461, 1356, 931, 803, 791, 744; ^1^H NMR (400 MHz, DMSO-*d_6_*) δ (ppm): 8.46 (s, 1H, C-H_arom_), 8.17 (d, *J* = 8.7 Hz, 1H, C-H_arom_), 8.10 (d, *J* = 8.7 Hz, 1H, C-H_arom_), 8.02 (t, *J* = 6.8 Hz, 1H, C-H_arom_), 7.94 (s, 1H, CH), 7.69–7.60 (m, 3H, 3 × C-H_arom_), 7.56 (d, *J* = 8.3 Hz, 1H, C-H_arom_), 7.28 (t, *J* = 7.7 Hz, 1H, C-H_arom_), 7.13 (t, *J* = 7.2 Hz, 1H, C-H_arom_), 6.87 (s, 1H, C_3_-H, indole), 3.93 (s, 3H, CH_3_); ^13^C NMR (100 MHz, DMSO-*d_6_*) δ (ppm): 146.6, 139.2, 135.3, 134.2, 133.1, 131.6, 131.1, 129.2, 129.1, 128.5, 128.3, 127.6, 127.2, 125.3, 123.4, 121.2, 120.7, 118.2, 110.9, 104.3, 102.5, 31.9. Anal. calcd for C_22_H_16_N_2_ (308.38) (%): C, 85.69; H, 5.23; N, 9.08. Found: C, 85.75; H, 5.31; N, 8.94.

#### 3.1.7. General Procedure for the Preparation of 2-(1-Acetyl-1*H*-indol-2-yl)-3-acrylonitriles **6a** and **6b** and 3-(4-Methoxyphenyl)-2-(1-(methylsulfonyl)-1*H*-indol-2-yl)acrylonitrile (**7**)

To a stirred solution of the appropriate 2-(1*H*-indol-2-yl)-3-acrylonitrile derivative (**2d** or **2l**) (0.75 mmol) in anhydrous dimethylformamide (5 mL) was added sodium hydride (36 mg, 0.9 mmol, 60% oil dispersion) in one portion at 0 °C. The reaction mixture was stirred for an additional 30 min at ambient temperature. Then, the reaction mixture was cooled to 0 °C and treated with acetyl chloride (71 mg, 64 µL, 0.9 mmol) or methanesulfonyl chloride (103 mg, 70 µL, 0.9 mmol). After stirring overnight at ambient temperature, the mixture was diluted with water and the resulting precipitate was collected by filtration, washed with water, and dried. Thus, the obtained crude products **6a**, **6b**, and **7** were purified on silica gel by column chromatography with dichloromethane as the eluent. In this manner, the following compounds were obtained.

*2-(1-Acetyl-1H-indol-2-yl)-3-(4-methoxyphenyl)acrylonitrile* (**6a**). Yield: 42% (beige solid); m.p. 119–122 °C; IR ν_max_ (KBr, cm^−1^): 3112, 3085, 3022, 2962, 2932, 2836, 2207, 1702, 1604, 1515, 1450, 1366, 1306, 1263, 1189, 1027, 837, 827, 740; ^1^H NMR (400 MHz, DMSO-*d_6_*) δ (ppm): 8.01 (d, *J* = 8.4 Hz, 1H, C-H_arom_), 7.94 (d, *J* = 8.9 Hz, 2H, 2 × C-H_arom_), 7.68 (d, *J* = 7.6 Hz, 1H, C-H_arom_), 7.60 (s, 1H, CH), 7.42 (t, *J* = 7.3 Hz, 1H, C-H_arom_), 7.33 (t, *J* = 7.5 Hz, 1H, C-H_arom_), 7.13 (d, *J* = 8.8 Hz, 2H, 2 × C-H_arom_), 7.05 (s, 1H, C_3_-H, indole), 3.86 (s, 3H, CH_3_), 2.80 (s, 3H, CH_3_); ^13^C NMR (100 MHz, DMSO-*d_6_*) δ (ppm): 170.4, 161.9, 144.8, 136.5, 135.4, 131.6 (two overlapping signals), 129.1, 126.3, 126.0, 124.0, 121.9, 118.0, 115.6, 115.1 (two overlapping signals), 113.3, 101.7, 56.0, 27.3. Anal. calcd for C_20_H_16_N_2_O_2_ (316.35) (%): C, 75.93; H, 5.10; N, 8.86. Found: C, 75.85; H, 4.88; N, 8.94.
*2-(1-Acetyl-1H-indol-2-yl)-3-[4-(dimethylamino)phenyl]acrylonitrile* (**6b**). Yield: 31% (yellow solid); m.p. 164–166 °C; IR ν_max_ (KBr, cm^−1^): 3115, 2899, 2817, 2204, 1692, 1612, 1579, 1524, 1450, 1367, 1303, 1169, 809, 761, 742; ^1^H NMR (400 MHz, DMSO-*d_6_*) δ (ppm): 8.04 (d, *J* = 8.2 Hz, 1H, C-H_arom_), 7.85 (d, *J* = 9.0 Hz, 2H, 2 × C-H_arom_), 7.65 (d, *J* = 7.6 Hz, 1H, C-H_arom_), 7.47 (s, 1H, CH), 7.39 (t, *J* = 7.2 Hz, 1H, C-H_arom_), 7.31 (t, *J* = 7.4 Hz, 1H, C-H_arom_), 6.97 (s, 1H, C_3_-H, indole), 6.83 (d, *J* = 9.0 Hz, 2H, 2 × C-H_arom_), 3.05 (s, 6H, 2 × CH_3_), 2.76 (s, 3H, CH_3_); ^13^C NMR (100 MHz, DMSO-*d_6_*) δ (ppm): 170.6, 152.5, 146.0, 136.7, 136.1, 131.6 (two overlapping signals), 129.1, 125.7, 124.0, 121.5, 120.8, 115.6, 112.6, 112.1 (two overlapping signals), 112.0, 96.5, 49.1 (two overlapping signals), 27.3; MS (ESI) *m*/*z*: 330 [M + H]^+^. Anal. calcd for C_21_H_19_N_3_O (329.40) (%): C, 76.57; H, 5.81; N, 12.76. Found: C, 76.65; H, 5.75; N, 12.82.
*3-(4-Methoxyphenyl)-2-(1-(methylsulfonyl)-1H-indol-2-yl)acrylonitrile* (**7**). Yield: 22% (white solid); m.p. 169–171 °C; IR ν_max_ (KBr, cm^−1^): 3109, 3075, 3003, 2972, 2922, 2214, 1608. 1512, 1369, 1167, 1070, 833, 773, 747, 556; ^1^H NMR (400 MHz, DMSO-*d_6_*) δ (ppm): 7.95–7.93 (m, 3H, 3 × C-H_arom_), 7.74–7.71 (m, 2H, C-H_arom_ + CH), 7.47 (t, *J* = 7.2 Hz, 1H, C-H_arom_), 7.39 (t, *J* = 7.5 Hz, 1H, C-H_arom_), 7.16–7.14 (m, 3H, 2 × C-H_arom_ + C_3_-H indole), 3.86 (s, 3H, CH_3_), 3.31 (s, 3H, CH_3_); ^13^C NMR (100 MHz, DMSO-*d_6_*) δ (ppm): 162.1, 147.0, 137.3, 135.7, 131.8 (two overlapping signals), 129.5, 126.3, 126.1, 124.8, 122.2, 118.1, 115.1 (two overlapping signals), 115.0, 114.0, 99.7, 56.0, 40.9. Anal. calcd for C_19_H_16_N_2_O_3_S (352.41) (%): C, 64.76; H, 4.58; N, 7.95. Found: C, 64.68; H, 4.64; N, 7.87.

### 3.2. Biology

#### 3.2.1. Evaluation of In Vitro Antiproliferative Activity

The in vitro anticancer assay was conducted at the National Cancer Institute (NCI) in Bethesda, USA against approximately 60 cancer cell lines [64,65,66,67].

The one-dose data were reported as a mean graph of the percent growth of treated cells. The number reported for the one-dose assay was the growth relative to the no-drug control and relative to the number of cells at time zero. This allowed detection of both growth inhibition (values between 0 and 100) and lethality (values less than 0). For example, a value of 100 meant no growth inhibition. A value of 40 would mean 60% growth inhibition. A value of 0 meant no net growth over the course of the experiment. A value of −40 would mean 40% lethality. A value of −100 meant all cells were dead.

The human tumor cell lines of the cancer screening panel were grown in RPMI 1640 medium containing 5% fetal bovine serum and 2 mM L-glutamine. For a typical screening experiment, 100 μL of cells was inoculated into 96-well microtiter plates at plating densities ranging from 5000 to 40,000 cells/well depending on the doubling time of the individual cell lines. After cell inoculation, the microtiter plates were incubated at 37 °C, under 5% CO_2_ and 95% air, and 100% relative humidity for 24 h prior to the addition of the tested compounds. After 24 h, two plates of each cell line were fixed in situ with TCA to represent a measurement of the cell population for each cell line at the time of sample addition (Tz). The tested compounds were solubilized in dimethyl sulfoxide at 400-fold the desired final maximum test concentration and stored frozen prior to use. At the time of compound addition, an aliquot of frozen concentrate was thawed and diluted to twice the desired final maximum test concentration with complete medium containing 50 μg/mL gentamicin. Additional 4-fold, 10-fold, or ½ log serial dilutions were made to provide a total of five compound concentrations plus the control. Aliquots of 100 μL of these different compound dilutions were added to the appropriate microtiter wells already containing 100 μL of medium, resulting in the required final compound concentrations. Following compound addition, the plates were incubated for an additional 48 h at 37 °C, under 5% CO_2_ and 95% air, and 100% relative humidity. For adherent cells, the assay was terminated by the addition of cold TCA. Cells were fixed in situ by the gentle addition of 50 μL of cold 50% (*w*/*v*) TCA (final concentration, 10% TCA) and incubated for 60 min at 4 °C. The supernatant was discarded, and the plates were washed five times with tap water and air dried. Sulforhodamine B (SRB) solution (100 μL) at 0.4% (*w*/*v*) in 1% acetic acid was added to each well, and plates were incubated for 10 min at room temperature. After staining, unbound dye was removed by washing five times with 1% acetic acid and the plates were air dried. Bound stain was subsequently solubilized with 10 mM Trizma Base, and the absorbance was measured using an automated plate reader at a wavelength of 515 nm. For cells in suspension, the methodology was the same except that the assay was terminated by fixing the settled cells at the bottom of the wells by gently adding 50 μL of 80% TCA (final concentration, 16% TCA). Using seven absorbance measurements [time zero, (Tz), control growth, (C), and growth in the presence of a test compound at five concentration levels (Ti)], the percentage growth was calculated at each of the drug concentration levels. Percentage growth was calculated as:

[(Ti − Tz)/(C − Tz)] × 100 for concentrations for which Ti >/= Tz

[(Ti − Tz)/Tz] × 100 for concentrations for which Ti < Tz

Three dose–response parameters were calculated for each tested compound. Growth inhibition of 50% (GI_50_) was calculated from [(Ti − Tz)/(C − Tz)] × 100 = 50, which refers to the compound concentration resulting in 50% reduction in the net protein increase (as measured by SRB staining) in control cells during the compound incubation. The compound concentration resulting in total growth inhibition (TGI) was calculated from Ti = Tz. The LC_50_ (concentration of compound resulting in 50% reduction in the measured protein at the end of the drug treatment as compared to that at the beginning) indicating a net loss of cells following treatment was calculated from [(Ti − Tz)/Tz] × 100 = −50. Values were calculated for each of these three parameters if the level of activity was reached; however, if the effect was not reached or was exceeded, the value for that parameter was expressed as greater or less than the maximum or minimum concentration tested. Furthermore, a mean graph midpoint (MG-MID) was calculated for each of the mentioned parameters, giving an averaged activity parameter over all cell lines. For calculation of the MG-MID, insensitive cell lines were included with the highest concentration tested.

#### 3.2.2. In Vitro Antimicrobial Activity

Antimicrobial activity was tested using the following reference strains: *Staphylococcus aureus* ATCC 6538, *Staphylococcus epidermidis* PCM 2118, *Escherichia coli* ATCC 11229, *Pseudomonas aeruginosa* ATCC 15442, *Enterococcus faecalis* ATCC 11420, and *Candida albicans* ATCC 10231. Antibacterial activity of selected compounds (**2i**, **2q**, **2s**, and **2x**) was evaluated against clinical *S. aureus* strains isolated from various clinical samples (nasal mucus, saliva, sputum, pus, and blood) derived from the Department of Oral Microbiology collection. Minimal inhibitory concentrations (MICs) for tested compounds were determined using a broth dilution method as recommended by the Clinical Laboratory Standards Institute (CLSI) guidelines [83]. Polypropylene 96-well plates with the investigated compounds serially diluted in Mueller Hinton Broth 2 (Sigma-Aldrich, St. Louis, MO, USA) (or in Sabouraud Dextrose Broth (BD Difco) for *C. albicans*) and initial inoculum 5 × 10^5^ CFU/mL were incubated at 37 °C for 18 h (or 24–48 h for *C. albicans*). MIC was taken as the lowest compound concentration at which observable growth was inhibited. Minimal bactericidal concentration (MBC) was determined in a sample taken from each test tube in which no growth was observed in the MIC assay. A loopful (10 µL) of the tested sample was transferred to Tryptic Soy Agar (TSA, BD Difco) (or Sabouraud Dextrose Agar (Sigma-Aldrich) for *C. albicans*) and incubated at 37 °C for 48 h. MBC was taken as the lowest concentration of tested compound that resulted in more than 99.9% reduction of the initial inoculum. Solutions of compounds were made fresh on the day of the assay. All experiments were performed in triplicate. The reference strains were stored at −80 °C in Tryptic Soy Broth (TSB, Oxoid, England) supplemented with 15% glycerol.

### 3.3. Computational Studies

#### 3.3.1. Preparation of Ligands and Proteins for Modeling

Crystal structures of the enzyme–DNA complexes were obtained from the Protein Data Bank [84]. In the study, the following proteins were used: caspase-3 from *Homo sapiens* (PDB code 2xyp) [72], caspase-9 from *H. sapiens* (PDB code 2ar9) [73], tubulin from *Ovis aries* (PDB code 5eyp) [74], penicillin-binding protein 4 from *E. coli* (PDB code 2ex8) [75], and *β*-lactamase from *E. coli* (PDB code 1fqg) [76]. The proteins were prepared using MAKE RECEPTOR software [85,86,87]. The pocket around the ligand bound in the crystal structure was generated automatically and was not adjusted, which resulted in grid boxes of various sizes (specifically 4946, 2022, 6206, 4292, and 4491 Ǻ for 2xyp, 2ar9, 5eyp, 2ex8, and 1fqg, respectively). A slow and effective “Molecular” method was used for “Cavity detection,” i.e., detection of binding sites. Outer and inner contours of the grid box were also calculated automatically using the “Balanced” settings for the “Site Shape Potential” calculation, which once more resulted in different outer contour sizes depending on the bound ligand, specifically 1645, 1219, 1339, 1562, and 1520Ǻ, respectively. The inner contours were disabled. No constraints for docking calculations were used.

The structures of compounds were prepared in SMILES notation. A library of conformers was generated with the OMEGA default settings, which resulted in a maximum of 200 conformers per ligand [87,88].

#### 3.3.2. Molecular Docking

The compounds were docked using the FRED algorithm [85,86]. The docking resolution was set to high while the other settings were set as default. Ten docking solutions were inspected visually and the best-ranked HYBRID-calculated conformations were used for analysis and representation. The docking protocols were validated by re-docking the co-crystallized ligands with RMSD values below 2 Å for each binding pocket.

#### 3.3.3. ADME/Drug-Likeness Calculation

The physicochemical, pharmacokinetic, and drug-likeness properties of compounds **2l**, **2x**, and **5a**–**d** were predicted using the SwissADME web tool and PreADMET server, which are available online [89,90].

## 4. Conclusions

A series of 2-(1*H*-indol-2-yl)-3-acrylonitriles **2a**–**x** and their derivatives **3**, **4a**–**c**, **5a**–**d**, **6a**–**b**, and **7** were synthesized and evaluated in terms of their anticancer and antimicrobial properties.

Analysis of the structure–activity relationship for the antiproliferative activity of all of the prepared compounds against a panel of approximately 60 human cancer cell lines revealed that heterocyclic replacement of the aromatic ring at position 3 of the acrylonitrile moiety led to a strong decrease in activity. The acrylonitrile moiety was required for cell growth inhibition because either replacing this moiety with an imino-acetonitrile group or its saturation yielded compounds with poor or lacking activity. Otherwise, the introduction of a methyl group at position 1 of the 2-(1*H*-indol-2-yl)-3-acrylonitrile scaffold improved antitumor potency. The 2-(1*H*-indol-2-yl)-3-acrylonitriles **2l** and **5a**–**d** were the most potent of all tested derivatives, exhibiting significant activity against the tumor cell lines investigated (full panel GI_50_ MD-MIG = 0.38–7.91 μM). Particularly, compound **5c** bearing a methyl group at position 1 of the indole ring and a 4-(dimethylamino)phenyl group at position 3 of the acrylonitrile moiety demonstrated optimal properties (full panel GI_50_ MD-MIG = 0.38 μM and TGI = 0.0866–5.06 μM against 13 cell lines of different cancer subpanels) and therefore may serve as a useful scaffold for further development of more potent antitumor agents.

Moreover, 2-(1*H*-indol-2-yl)-3-acrylonitriles **2a**–**c** and **2e**–**x** were tested against Gram-positive and Gram-negative bacterial strains, namely *Staphylococcus aureus*, *Staphylococcus epidermi*, *Enterococcus faecalis*, *Escherichia coli*, and *Pseudomonas aeruginosa*, as well as fungal species *Candida albicans*. The majority of the tested 2-(1*H*-indol-2-yl)-3-acrylonitriles were found to be rather inactive; however, compounds **2i**, **2q**, **2s**, and **2x** showed promising potential against some bacterial species. An interesting compound is 2-(1*H*-indol-2-yl)-3-(1*H*-pyrrol-2-yl)acrylonitrile (**2x**), which exhibited relatively high antibacterial activity against all Gram-positive bacteria tested (MIC = 8–32 μg/mL, MBC = 32 μg/mL). Furthermore, it was the only compound among those tested that showed activity against Gram-negative bacteria, e.g., *E. coli* (MIC = MBC = 32 μg/mL). The analogue **2x** also effectively inhibited the growth of various strains of *S. aureus* isolated from clinical specimens (MIC values of 16 μg/mL). In addition, derivative **2x** was found to have pronounced antifungal activity against *C. albicans* (MIC = 4 μg/mL, MFC = 8 μg/mL). Based on these findings, compound **2x** seems to be leading compound for further development as an antimicrobial agent.

The results of the docking studies suggested that, like other heteroaryl-acrylonitriles, the obtained compounds may exert their cancer cell growth inhibitory effects through interaction with tubulin in the colchicine-binding site and/or apoptotic caspase-3 as well as caspase-9, whereas their antibacterial activity may be related to interaction with PBP4 and/or *β*-lactamase. However, it is too early to speculate on the mode of action of these indole-acrylonitriles. For example, a number of mechanisms could be responsible for their antitumor effects, e.g., specific interaction with cellular receptors and enzymes or 1,4-nucleophilic addition of thiol to the acrylonitrile double bond [49,50]. As Michael acceptors, acrylonitriles react in vitro or in vivo with sulfur-containing nucleophiles such as glutathione (GSH) and proteins. It is worth noting that acrylonitrile-based Michael acceptors activated by aryl or heteroaryl electron-withdrawing groups have been reported as reversible, cysteine-targeted kinase inhibitors [91]. It is well known that such covalent, electrophilic “warheads” targeting cysteine residues constitute a promising approach in drug development [92]. Therefore, it remains a challenge to explore in depth the mode of action and the pharmacodynamic features of these compounds, which will be the next goal of this project.

According to the predicted ADME/drug-likeness properties, the most active compounds **2l**, **2x**, and **5a**–**d** were shown to be drug-likeness molecules.

## Figures and Tables

**Table 1:** Mean growth (%), growth inhibition percent (%GI), and lethality values for the most sensitive cell lines among approximately NCI-60 human cancer cell lines after treatment with the tested compounds at 10 μM ^a^.

Compound	Mean Growth	Most Sensitive Cell Line	Growth Inhibition Percent (%GI) ^b^/Lethality ^c^
**2a**	85.56	T-47D (*breast cancer*)	59.79
**2b**	49.80	MDA-MB-435 (*melanoma*)	−5.10 ^c^
**2c**	78.15	K-562 (*leukemia*)	72.20
**2d**	30.17	MDA-MB-435 (*melanoma*)	−32.56 ^c^
**2e**	54.83	MDA-MB-435 (*melanoma*)	−28.75 ^c^
**2f**	66.94	MDA-MB-435 (*melanoma*)	87.84
**2g**	93.62	A549/ATCC (*non-small cell lung cancer*)	39.97
**2h**	84.56	CCRF-CEM (*leukemia*)	−4.12 ^c^
**2i**	84.18	HOP-92 (*non-small cell lung cancer*)	46.93
**2j**	93.18	MCF7 (*breast cancer*)	36.48
**2k**	91.86	CCRF-CEM (*leukemia*)	−42.43 ^c^
**2l**	26.05	MDA-MB-435 (*melanoma*)	−25.37 ^c^
**2m**	53.62	MDA-MB-435 (*melanoma*)	98.20
**2n**	73.11	K-562 (*leukemia*)	71.26
**2o**	85.25	T-47D (*breast cancer*)	43.76
**2p**	36.99	MDA-MB-435 (*melanoma*)	−18.97 ^c^
**2q**	83.41	MCF7 (*breast cancer*)	79.89
**2r**	88.94	CCRF-CEM (*leukemia*)	53.33
**2s**	83.36	T-47D (*breast cancer*)	84.11
**2t**	88.41	SNB-75 (*CNS cancer*)	56.11
**2u**	92.81	MCF7 (*breast cancer*)	67.84
**2v**	95.03	SNB-75 (*CNS cancer*)	46.36
**2w**	91.64	SNB-75 (*CNS cancer*)	59.89
**2x**	66.37	MDA-MB-468 (*breast cancer*)	−3.15 ^c^
**3**	90.03	UO-31 (*renal cancer*)	45.43
**4a**	87.87	CCRF-CEM (*leukemia*)	−27.23 ^c^
**4b**	94.94	UO-31 (*renal cancer*)	36.32
**4c**	89.04	CNB-75 (*CNS cancer*)	60.83
**5a**	32.33	OVCAR-3 (*ovarian cancer*)	−12.57 ^c^
**5b**	24.19	MDA-MB-435 (*melanoma*)	−49.06 ^c^
**5c**	25.80	MDA-MB-435 (*melanoma*)	−26.92 ^c^
**5d**	25.56	SK-MEL-5 (*melanoma*)	−26.68 ^c^
**6a**	81.55	MDA-MB-435 (*melanoma*)	90.31
**6b**	33.80	SNB-75 (*CNS cancer*)	−6.19 ^c^
**7**	82.53	MDA-MB-435 (*melanoma*)	93.95

^a^ Data obtained from NCI-60 DTP human tumor cell line screen [67]; ^b^ %GI (growth inhibition percent) = 100 − GP (growth percent); ^c^ negative values mean lethality of the respective cancer cell line.

**Table 2:** Overview of the results of the in vitro antitumor screening for compounds **2l** and **5a**–**d** ^a^.

		Compounds									
		2l		5a		5b		5c		5d	
Panel Name	Cell Name	GI_50_^b^	TGI ^c^	GI_50_^b^	TGI ^c^	GI_50_^b^	TGI ^c^	GI_50_^b^	TGI ^c^	GI_50_^b^	TGI ^c^
*Leukemia*	CCRF-CEM	0.813	14.3	0.373	>100	0.333	>100	0.213	>100	0.218	>100
	HL-60(TB)	0.455	>100	0.305	6.64	0.217	0.582	0.103	0.555	0.179	0.703
	K-562	−	−	0.244	>100	0.216	>100	0.0364	>100	0.0406	>100
	MOLT-4	0.906	>100	0.507	>100	0.417	16.2	0.338	>100	0.446	>100
	RPMI-8226	0.63	4.91	0.39	>100	0.395	>100	0.296	−	0.285	>100
	SR	0.497	>100	0.269	>100	0.179	0.94	0.0357	1.81	0.0578	>100
*Non-small cell lung cancer*	A549/ATCC	1.55	>100	0.438	>100	0.525	>100	0.159	>100	0.174	>100
	EKVX	2.94	>100	0.549	>100	0.821	>100	0.64	>100	0.442	>100
	HOP-62	1.16	4.54	0.4	>100	0.411	>100	0.206	>100	0.387	>100
	HOP-92	1.42	5.53	0.61	>100	0.633	>100	0.189	5.29	0.0395	>100
	NCI-H226	1.80	−	0.753	>100	4.26	>100	5.06	>100	0.325	>100
	NCI-H23	3.21	>100	0.536	>100	0.739	>100	0.734	>100	0.361	>100
	NCI-H322M	2.88	>100	0.722	>100	0.57	>100	0.671	>100	−	>100
	NCI-H460	0.476	>100	0.366	>100	0.397	21.3	0.307	−	0.182	>100
	NCI-H522	0.542	>100	0.259	4.57	0.19	0.554	0.0244	0.0866	0.0277	−
*Colon cancer*	COLO 205	0.566	7.39	0.405	>100	0.321	1.04	0.134	0.498	0.29	>100
	HCC-2998	6.6	>100	0.522	>100	1.05	5.86	0.323	>100	0.308	>100
	HCT-116	0.456	>100	0.367	>100	0.409	>100	0.0954	>100	0.042	>100
	HCT-15	0.509	>100	0.328	>100	0.359	>100	0.0587	>100	0.064	>100
	HT29	0.375	15.9	0.351	>100	0.336	11.6	0.0491	1.43	0.153	−
	KM12	0.495	>100	0.431	>100	0.504	>100	0.285	>100	0.0922	>100
	SW-620	0.39	61.9	0.389	>100	0.366	>100	0.0825	>100	0.1	>100
*CNS cancer*	SF-268	2.52	>100	4.02	>100	1.19	>100	1.9	>100	−	>100
	SF-295	0.61	7.33	0.285	1.46	0.403	7.94	0.0828	1.28	0.0599	−
	SF-539	1.97	35.1	0.313	1.09	0.283	0.891	0.17	0.548	0.0613	0.552
	SNB-19	2.23	>100	0.448	>100	0.487	>100	0.344	>100	0.207	>100
	SNB-75	1.31	4.59	0.232	−	0.15	0.514	0.0193	0.196	0.0554	85.9
	U251	0.913	18.6	0.389	>100	0.551	>100	0.299	>100	0.167	>100
*Melanoma*	LOX IMVI	0.692	>100	0.532	>100	0.654	>100	0.471	>100	0.0705	>100
	MALME-3M	2.32	>100	−	>100	0.36	>100	0.0725	>100	−	>100
	M14	0.551	>100	0.203	−	0.338	>100	0.0855	−	0.0666	>100
	MDA-MB- 435	0.228	0.641	0.0726	0.254	0.153	0.403	0.0285	−	0.0318	0.103
	SK-MEL-2	0.454	>100	−	>100	0.417	>100	0.328	>100	72.9	>100
	SK-MEL-28	2.63	>100	−	>100	6.96	−	−	>100	16.6	>100
	SK-MEL-5	1.36	32.8	0.717	>100	0.446	14.3	0.326	−	0.455	>100
	UACC-257	4.86	>100	>100	>100	0.679	>100	2.98	>100	>100	>100
	UACC-62	0.679	>100	0.34	>100	0.54	>100	0.163	>100	0.0436	>100
*Ovarian cancer*	IGROV1	1.6	>100	0.555	>100	0.505	>100	0.165	>100	0.15	>100
	OVCAR-3	0.409	4.06	0.333	2.24	0.302	0.955	0.142	0.663	0.179	−
	OVCAR-4	3.75	>100	−	>100	0.67	>100	0.521	>100	>100	>100
	OVCAR-5	3.80	>100	0.607	>100	0.995	>100	0.428	>100	0.329	>100
	OVCAR-8	2.35	>100	0.584	>100	0.577	>100	0.338	>100	0.392	>100
	NCI/ADR- RES	0.49	>100	0.379	>100	0.317	8.24	0.0736	>100	0.0607	>100
	SK-OV-3	2.28	>100	−	>100	0.517	>100	0.377	>100	−	>100
*Renal cancer*	786-0	2.52	>100	0.676	>100	−	−	−	−	0.306	>100
	A498	0.391	6.61	0.235	0.816	0.277	0.867	0.0795	0.938	0.03	0.418
	ACHN	2.42	>100	0.763	>100	0.629	>100	0.39	>100	0.0605	>100
	CAKI-1	1.6	>100	0.329	>100	0.444	>100	0.0736	>100	0.0545	>100
	RXF 393	−	−	0.243	−	0.217	0.751	0.133	0.549	0.0475	−
	SN12C	2.37	>100	0.69	>100	0.508	>100	0.392	>100	0.362	>100
	TK-10	2.66	>100	>100	>100	0.902	>100	−	>100	>100	>100
	UO-31	2.01	>100	0.792	>100	0.514	>100	0.786	>100	0.0731	>100
*Prostate cancer*	PC-3	1.15	31.5	0.431	>100	0.432	>100	0.166	>100	0.2	>100
	DU-145	3.41	35.4	0.469	>100	0.529	>100	0.488	>100	0.45	>100
*Breast cancer*	MCF7	0.416	>100	0.356	>100	0.317	>100	0.0309	>100	0.0692	>100
	MDA-MB-231/ATCC	1.63	>100	0.713	>100	0.615	>100	0.428	>100	0.406	>100
	HS 578T	1.63	9.49	0.484	>100	0.375	>100	0.427	>100	0.201	>100
	BT-549	3.86	>100	0.65	7.12	0.704	−	0.806	>100	0.251	−
	T-47D	0.585	>100	0.631	>100	0.31	>100	0.0764	>100	>100	>100
	MDA-MB-468	0.373	4.2	0.406	>100	0.236	0.826	0.0384	0.501	0.55	>100

^a^ Data obtained from the NCI in vitro disease-oriented human tumor cell lines screen in μM [67]; ^b^ GI_50_—the molar concentration that inhibits 50% net cell growth; ^c^ TGI—the molar concentration leading to total growth inhibition;—not tested.

**Table 3:** GI_50_ mean-graph midpoint (MG-MID) ^a^ of subpanel cancer cell lines for compounds **2l** and **5a**–**d**.

Subpanel Cancer Cell Line	Compounds				
	2l	5a	5b	5c	5d
*Leukemia*	0.66	0.35	0.29	0.17	0.20
*Non-small cell lung cancer*	1.78	0.51	0.95	0.89	0.24
*Colon cancer*	1.34	0.40	0.48	0.15	0.15
*CNS cancer*	1.59	0.95	0.51	0.47	0.11
*Melanoma*	1.53	>16.98	1.17	0.56	23.77
*Ovarian cancer*	2.10	0.49	0.55	0.29	16.85
*Renal cancer*	2.00	>12.97	0.50	0.31	12.62
*Prostate cancer*	2.28	0.45	0.48	0.33	0.33
*Breast cancer*	1.42	0.54	0.43	0.30	16.91
*Full panel MG-MID ^b^*	1.63	>3.74	0.60	0.38	>7.91

^a^ Median value calculated based on the data from the NCI in vitro cytotoxicity screening for each cancer type cell line; ^b^ GI_50_ full panel mean-graph midpoint (MG-MID) is the average GI_50_ value against all cancer cell lines.

**Table 4:** Minimal inhibitory concentration (MIC) and minimal bactericidal concentration (MBC) of **2a**–**x** against Gram-positive and Gram-negative reference bacteria as well as minimal fungicidal concentration (MFC) against *Candida albicans*.

Compound	Strain											
	*S. aureus* ATCC 6538		*S. epidermidis* PCM 2118		*E. faecalis* ATCC 11420		*E. coli* ATCC 11229		*P. aeruginosa* ATCC 15442		*C. albicans* ATCC 10231	
	MIC	MBC	MIC	MBC	MIC	MBC	MIC	MBC	MIC	MBC	MIC	MFC
	(μg/mL)											
**2a**	>256	>256	>256	>256	>256	>256	>256	>256	>256	>256	128	256
**2b**	>256	>256	>256	>256	>256	>256	>256	>256	>256	>256	128	256
**2c**	128	>256	128	>256	>256	>256	>256	>256	>256	>256	128	128
**2e**	>256	>256	>256	>256	>256	>256	>256	>256	>256	>256	256	256
**2f**	>256	>256	>256	>256	>256	>256	>256	>256	>256	>256	256	256
**2g**	128	>256	128	>256	>256	>256	>256	>256	>256	>256	128	128
**2h**	>256	>256	128	256	>256	>256	>256	>256	>256	>256	128	256
**2i**	16	16	8	16	>256	>256	>256	>256	>256	>256	128	256
**2j**	>256	>256	>256	>256	>256	>256	>256	>256	>256	>256	256	256
**2k**	128	>256	128	>256	>256	>256	>256	>256	>256	>256	256	256
**2l**	>256	>256	>256	>256	>256	>256	>256	>256	>256	>256	128	256
**2n**	64	128	128	256	>256	>256	>256	>256	>256	>256	256	256
**2o**	>256	>256	128	256	>256	>256	>256	>256	>256	>256	256	256
**2p**	128	>256	128	>256	>256	>256	>256	>256	>256	>256	>256	>256
**2q**	8	8	16	16	>256	>256	>256	>256	>256	>256	128	256
**2r**	>256	>256	>256	>256	>256	>256	>256	>256	>256	>256	128	256
**2s**	16	32	32	32	>256	>256	>256	>256	>256	>256	16	128
**2t**	>128	>128	>128	>128	>128	>128	>128	>128	>128	>128	>128	>128
**2u**	>128	>128	>128	>128	>128	>128	>128	>128	>128	>128	>128	>128
**2v**	>128	>128	>128	>128	>128	>128	>128	>128	>128	>128	>128	>128
**2w**	>128	>128	>128	>128	>128	>128	>128	>128	>128	>128	>128	>128
**2x**	8	32	8	32	32	32	32	32	>256	>256	4	8

**Table 5:** Minimal inhibitory concentration (MIC) and minimal bactericidal concentration (MBC) of **2i**, **2q**, **2s**, and **2x** against clinical *S. aureus* strains.

Compound	Strain													
	79		124		128		143		177		220		244	
	MIC	MBC	MIC	MBC	MIC	MBC	MIC	MBC	MIC	MBC	MIC	MBC	MIC	MBC
	(μg/mL)													
**2i**	>128	>128	>128	>128	>128	>128	>128	>128	>128	>128	>128	>128	>128	>128
**2q**	>256	>256	>256	>256	>256	>256	>256	>256	>256	>256	>256	>256	>256	>256
**2s**	>128	>128	>128	>128	>128	>128	>128	>128	>128	>128	>128	>128	>128	>128
**2x**	16	64	16	32	16	32	16	32	16	32	16	32	16	32

**Table 6:** Minimal inhibitory concentration (MIC) and minimal bactericidal concentration (MBC) of **2x** against clinical methicillin-sensitive *S. aureus* (MSSA) and methicillin-resistant *S. aureus* (MRSA) strains.

Strain	MIC	MBC
	(μg/mL)	
	Compound 2x	
MSSA		
*S. aureus 1*	16	64
*S. aureus 2*	16	64
*S. aureus 3*	16	64
*S. aureus 4*	16	64
*S. aureus 5*	16	64
MRSA		
*S. aureus 6*	16	64
*S. aureus 7*	16	64
*S. aureus 8*	16	64
*S. aureus 9*	16	64
*S. aureus 10*	16	64

**Table 7:** Predicted physicochemical, pharmacokinetic, and drug-likeness properties of compounds **2l**, **2x**, and **5a**–**d**.

	Physicochemical Properties					Lipophilicity	Water Solubility	Pharmacokinetics		Drug–Likeness	
	mol. wt. (g/mol)	ROTB (n)	HBA (n)	HBD (n)	TPSA	CLogP o/w	Solubility Class	GI Absorption	BBB Permeant	Lipinski Filter	BS
Rule	<500	<10	<10	<5	-	<5	-	-	-	-	-
**2l**	287.36	3	1	1	42.82	3.49	Soluble (m)	High	Yes	Yes (0)	0.55
**2x**	233.27	2	1	2	55.37	2.61	Soluble	High	Yes	Yes (0)	0.55
**5a**	272.34	2	1	0	28.72	3.83	Soluble (m)	High	Yes	Yes (0)	0.55
**5b**	288.34	3	2	0	37.95	3.50	Soluble (m)	High	Yes	Yes (0)	0.55
**5c**	301.38	3	1	0	31.96	3.51	Soluble (m)	High	Yes	Yes (0)	0.55
**5d**	308.38	2	1	0	28.72	4.41	Soluble (m)	High	Yes	Yes (0)	0.55

mol. wt.—molecular weight; n—number, ROTB—rotable bonds; HBA—hydrogen bond acceptors; HBD—hydrogen bond donors; TPSA—topological polar surface area calculated in Å^2^; CLogPo/w—consensus logarithm of partition coefficient between *n*-octanol and water; m—moderate; Lipinski filter with number of violations in bracket; GI—gastrointestinal absorption; BBB—blood–brain barrier; BA—bioavailability score.

## Data Availability

Data are contained within the article and Supplementary Materials.

## Supplementary Materials

CCDC 2130049 contains the supplementary crystallographic data for this paper. These data can be obtained free of charge via http://www.ccdc.cam.ac.uk/conts/retrieving.html (or from the CCDC, 12 Union Road, Cambridge CB2 1EZ, UK; Fax: +44-1223-336033; E-mail: deposit@ccdc.cam.ac.uk) (accessed on 1 January 2023). The following supporting information can be downloaded at: https://www.mdpi.com/article/10.3390/ph16070918/s1, Table S1: FRED Chemgauss4 scores of the docked ligands **1**, **2a**–**x**, **3**, **4a**–**c**, **5a**–**d**, **6a**–**b**, and **7**; Table S2: Two-dimensional diagrams of interactions created by compounds **2a** and **5a**–**d** in the active site of caspase-3 (2xyp) generated by BIOVA Discovery Studio Visualizer and Pose View; Table S3: Two-dimensional diagrams of interactions created by compounds **2l** and **5a**–**d** in the active site of caspase-9 (2ar9) generated by BIOVA Discovery Studio Visualizer and Pose View; Table S4: Two-dimensional diagrams of interactions created by compounds **2l** and **5a**–**d** in the active site of tubulin (5eyp) generated by BIOVA Discovery Studio Visualizer and Pose View; Table S5: Two-dimensional diagrams of interactions created by compound **2x** in the active site of penicillin-binding protein 4 (2ex8) generated by BIOVA Discovery Studio Visualizer and Pose View; Table S6. Two-dimensional diagrams of interactions created by compound **2x** in the active site of β-lactamase (1fqg) generated by BIOVA Discovery Studio Visualizer and Pose View; Table S7: Predicted physicochemical, pharmacokinetic, and drug-likeness properties of compounds **2l**, **2x**, and **5a**–**d;** Table S8: Predicted human intestinal absorption, Caco-2 cell and MDCK cell permeabilities for compounds **2l**, **2x**, and **5a**–**d**.
