# Supplementary material for: Indole-Acrylonitrile Derivatives as Potential Antitumor and Antimicrobial Agents—Synthesis, In Vitro and In Silico Studies
Source: Pharmaceuticals (Basel). 2023 Jun 22;16(7):918. doi: 10.3390/ph16070918 (PMC10386429; doi:10.3390/ph16070918)
Supplement: Supplementary file 1 [file pharmaceuticals-16-00918-s001.zip › pharmaceuticals-2417453-supplementary.pdf]

Supplementary Materials

Indole-acrylonitrile derivatives as potential antitumor and antimicrobial agents – synthesis, *in vitro* and *in silico* studies

Anita Kornicka [1*], Karol Gzella [1], Katarzyna Garbacz [2], Małgorzata Jarosiewicz [2], Maria Gdaniec [3], Joanna Fedorowicz [1], Łukasz Balewski [1], Jakub Kokoszka [1], Anna Ordyszewska [4]

[1] Department of Chemical Technology of Drugs, Faculty of Pharmacy, Medical University of Gdansk, 80-416 Gdansk, Poland

[2] Department of Oral Microbiology, Medical Faculty, Medical University of Gdansk, 80-416 Gdansk, Poland

[3] Faculty of Chemistry, Adam Mickiewicz University, 61-614 Poznań, Poland

[4] Department of Inorganic Chemistry, Faculty of Chemistry and Advanced Materials Centers, Gdańsk University of Technology, Narutowicza 11/12, 80-233 Gdansk, Poland

[*]Correspondence: anita.kornicka@gumed.edu.pl.

Table of contents:

1. **Docking results**
   Table S1. FRED Chemgauss4 scores of the docked ligands **1**, **2a-x**, **3**, **4a-c**, **5a-d**, **6a-b** and **7.** **S2**
   Table S2. The 2D diagrams of interactions created by compounds **2l** and **5a-d** in the active site of caspase-3 (2xyp) generated by BIOVA Discovery Studio Visualizer and Pose View. **S3**
   Table S3. The 2D diagrams of interactions created by compounds **2l** and **5a-d** in the active site of caspase-9 (2ar9) generated by BIOVA Discovery Studio Visualizer and Pose View. **S5**
   Table S4. The 2D diagrams of interactions created by compounds **2l** and **5a-d** in the active site of tubulin (5eyp) generated by BIOVA Discovery Studio Visualizer and Pose View. **S7**
   Table S5. The 2D diagrams of interactions created by compounds **2x** in the active site of penicillin-binding protein 4 (2ex8) generated by BIOVA Discovery Studio Visualizer and Pose View. **S9**
   Table S6. The 2D diagrams of interactions created by compounds **2x** in the active site of β-lactamase (1fqg) generated by BIOVA Discovery Studio Visualizer and Pose View. **S9**
2. **Calculated ADME/drug-likeness profiles**
   Tables S7. Predicted physicochemical, pharmacokinetic and drug-likeness properties of compounds **2l**, **2x**, **5a-d.** **S10**
   Table S8. Predicted human intestinal absorption, Caco-2 cell and MDCK cell permeabilities for compounds **2l**, **2x**, **5a-d.** **S11**

Table S1. FRED Chemgauss4 scores of the docked ligands **1**, **2a-x**, **3**, **4a-c**, **5a-d**, **6a-b** and **7.**

FRED Chemgauss4 score					
**Compd**	**caspase-3 (2xyp)**	**caspase-9 (2ar9)**	**tubulin (5eyp)**	**penicillin-binding protein 4 (2ex8)**	**β-lactamase (1fqg)**
**1**	-5.23302	-5.08912	-8.9825	-5.27782	-6.51337
**2a**	-5.90363	-3.884	-10.8717	-5.17229	-8.23318
**2b**	-5.08525	-3.37537	-11.6776	-5.77427	-8.2824
**2c**	-5.60849	-3.65052	-11.4421	-5.0086	-8.18364
**2d**	-4.94597	-3.66019	-11.2076	-4.86242	-8.06483
**2e**	-6.27092	-3.58057	-10.6082	-5.30321	-8.12786
**2f**	-5.47292	-4.32452	-11.6918	-4.40842	-7.48552
**2g**	-5.51387	-3.34911	-11.1683	-5.73747	-7.95726
**2h**	-5.67204	-3.7449	-11.1333	-5.90206	-9.63335
**2i**	-5.67204	-3.7449	-11.1333	-5.90206	-9.63335
**2j**	-5.34182	-3.86886	-11.1676	-5.44939	-7.9373
**2k**	-5.11492	-3.82303	-10.779	-5.61474	-8.12498
**2l**	-5.54945	-3.57143	-11.4581	-5.12205	-8.33456
**2m**	-5.85053	-3.3784	-10.0974	-4.70873	-7.88071
**2n**	-4.71896	-3.80598	-10.2373	-4.84896	-7.80353
**2o**	-6.20214	-3.50577	-13.2754	-5.65625	-9.23724
**2p**	-6.61627	-3.47568	-12.2016	-5.56695	-8.22868
**2q**	-5.96685	-3.61183	-11.7359	-5.3542	-9.61483
**2r**	-6.7915	-4.39583	-11.7718	-5.20843	-7.84654
**2s**	-5.65366	-3.97643	-11.2263	-5.48613	-8.53547
**2t**	-5.86027	-3.8612	-10.9212	-5.38187	-8.18399
**2u**	-6.16928	-4.19446	-11.1868	-5.13292	-8.16696
**2v**	-5.27053	-3.8119	-11.1987	-4.94718	-8.15649
**2w**	-6.21192	-4.00936	-11.1138	-4.90712	-8.22966
**2x**	-6.20929	-6.6242	-10.2871	-5.99989	-7.75472
**3**	-6.48325	-3.37125	-9.97834	-4.45381	-8.18494
**4a**	-5.6432	-4.20064	-12.4149	-6.23501	-8.78394
**4b**	-5.85867	-3.97765	-11.548	-6.40248	-8.57117
**4c**	-6.31567	-4.53341	-12.3295	-6.87913	-8.76742
**5a**	-4.98728	-2.59772	-12.4227	-4.76406	-7.88166
**5b**	-4.92247	-2.4134	-12.3072	-4.77243	-7.60658
**5c**	-5.32272	-2.35997	-11.5326	-4.13846	-8.25605
**5d**	-6.09417	-3.05058	-13.3035	-5.41821	-8.54794
**6a**	-5.55541	-2.59091	-11.8886	-4.51006	-8.18193
**6b**	-5.98233	-3.47475	-11.6401	-4.3311	-7.96514
**7**	-4.87739	-2.1102	-12.0286	-4.53795	-7.71406
**original ligand***	-5.14153	-7.50372	-17.5854	-5.65605	-9.54773

*Phenylmethyl *N*-[(2S)-4-chloro-3-okso-1-phenyl-butan-2-yl]carbamate, D-maleate and colchicine (*N*-[(7S)-1,2,3,10-tetramethoxy-9-oxo-6,7-dihydro-5*H*-benzo[*d*]heptalen-7-yl]-ethanamide) were used as original ligands for 2xyp, 2ar9 and 5eyp, respectively, whereas penicillin G was used as the original ligand for 2ex8 and 1fqg.

Table S2. The 2D diagrams of interactions created by compounds **2l** and **5a-d** in the active site of caspase-3 (2xyp) generated by BIOVA Discovery Studio Visualizer and Pose View.

<table>
<tr><th>Compd</th><th>Discovery Studio</th><th>PoseView</th></tr>
<tr><td>2l</td><td>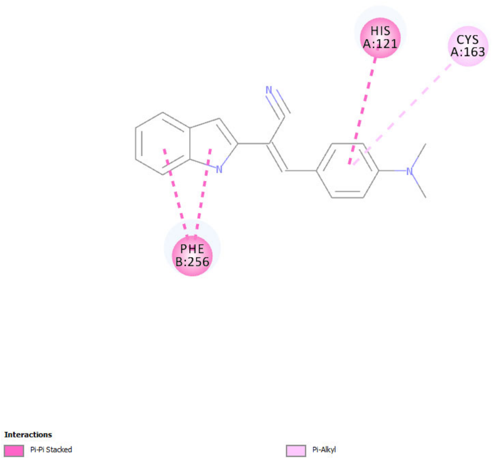
</td><td>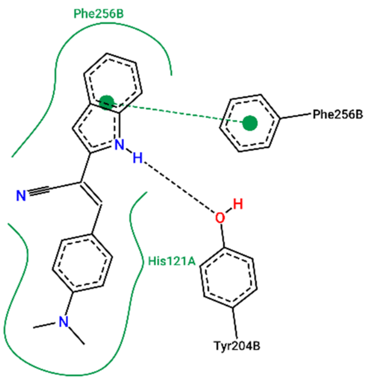
</td></tr>
<tr><td>5a</td><td>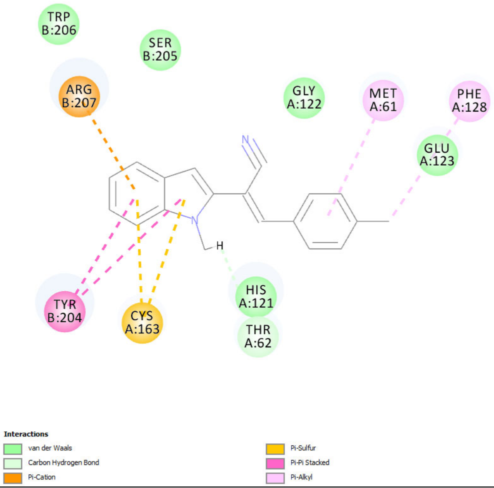
</td><td>No interactions found by the PoseView interaction model</td></tr>
<tr><td>5b</td><td>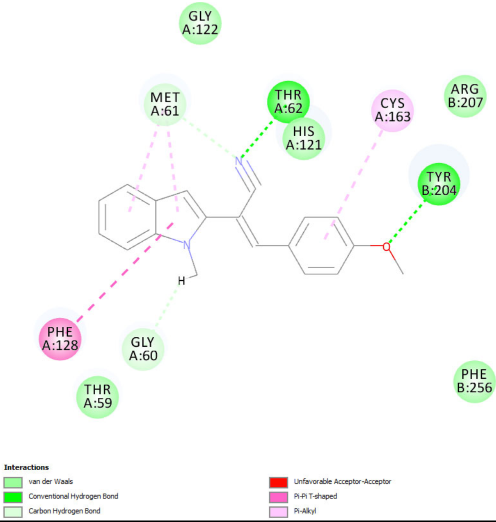
</td><td>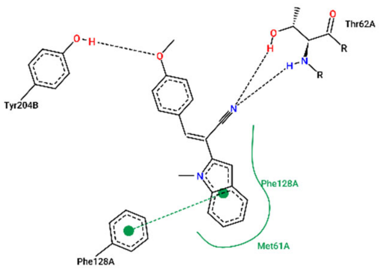
</td></tr>
</table>

Table S2. Continued

<table>
<tr><th>Compd</th><th>Discovery Studio</th><th>PoseView</th></tr>
<tr><td>5c</td><td>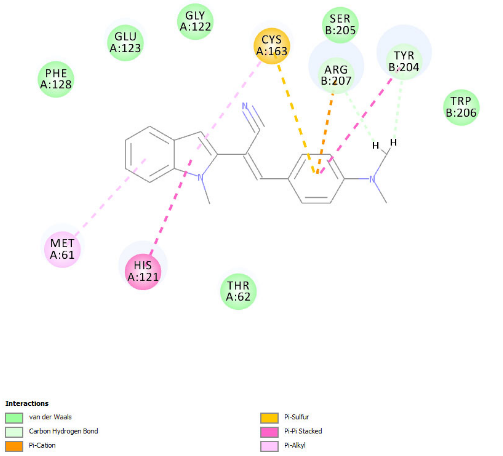
</td><td>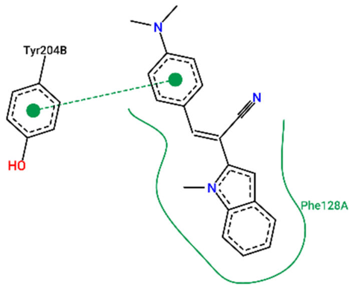
</td></tr>
<tr><td>5d</td><td>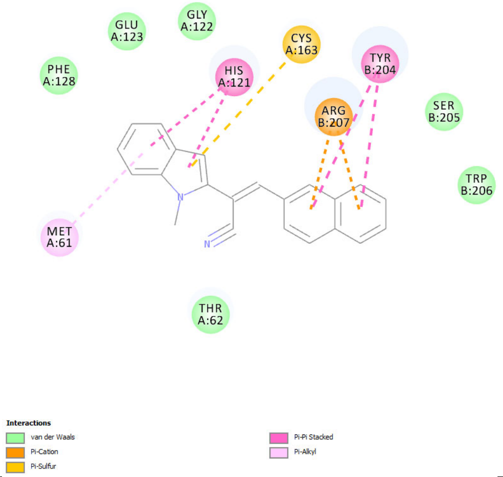
</td><td>No interactions found by the PoseView interaction model</td></tr>
</table>

Table S3. The 2D diagrams of interactions created by compounds **2l** and **5a-d** in the active site of caspase-9 (2ar9) generated by BIOVA Discovery Studio Visualizer and Pose View.

Compd	Discovery Studio	PoseView
**2l**	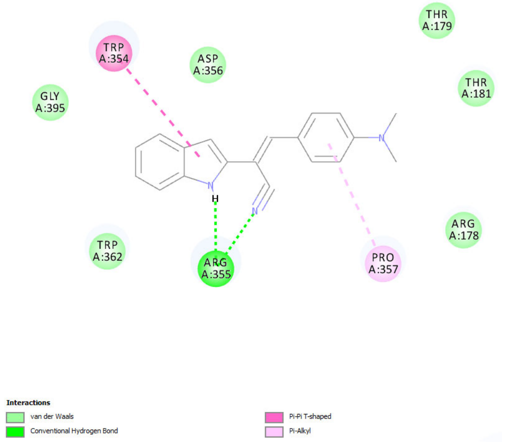	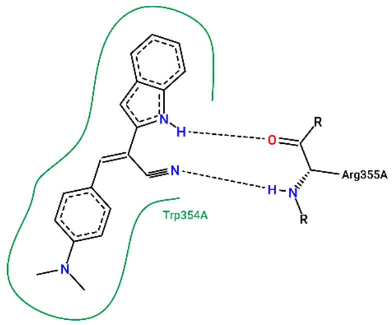
**5a**	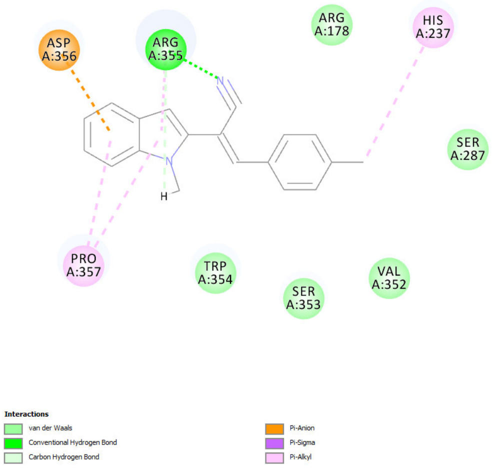	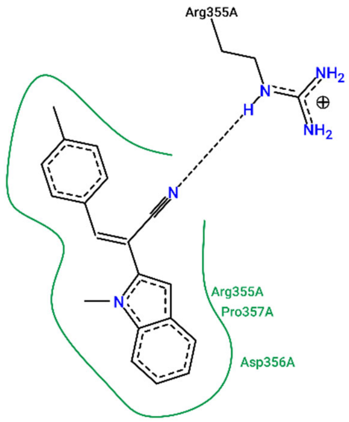
**5b**	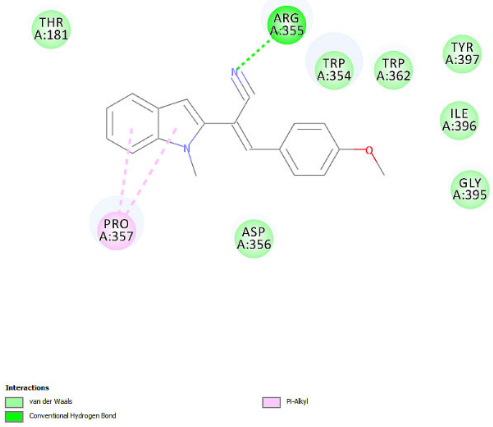	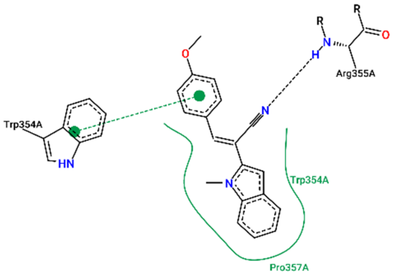

Table S3. Continued

Compd	Discovery Studio	PoseView
**5c**	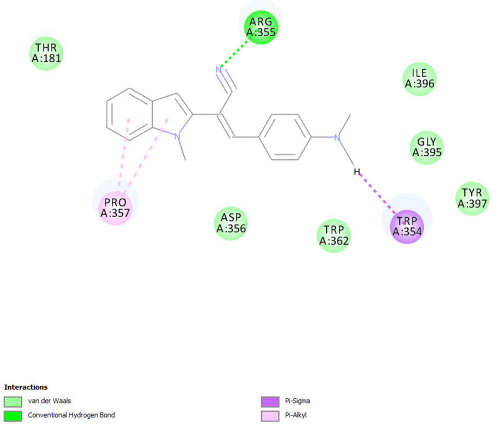	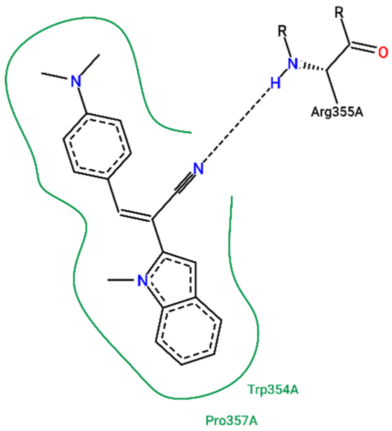
**5d**	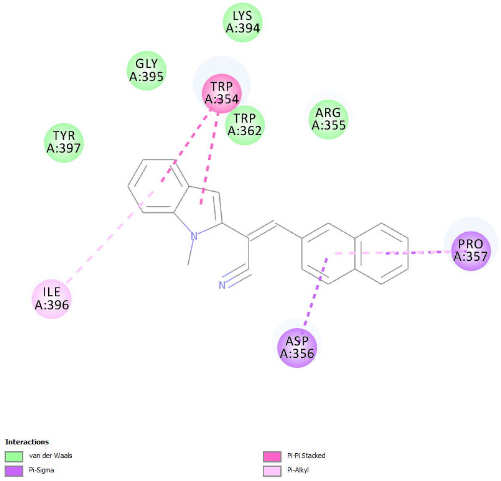	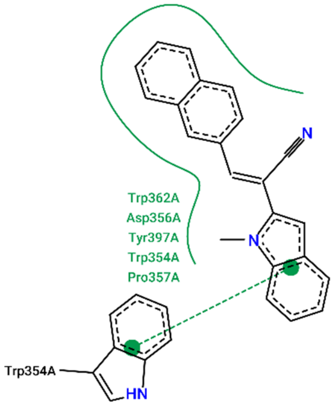

Table S4. The 2D diagrams of interactions created by compounds **2l** and **5a-d** in the active site of tubulin (5eyp) generated by BIOVA Discovery Studio Visualizer and Pose View.

Compd	Discovery Studio	PoseView
**2l**	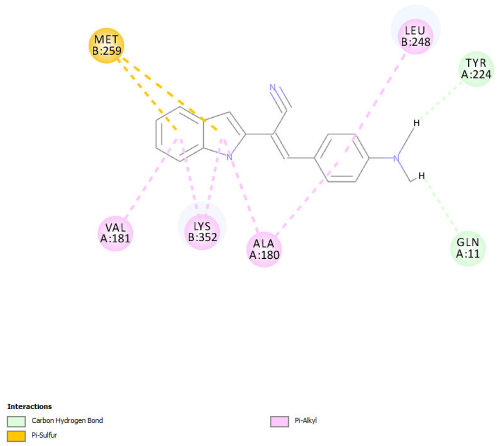	No interactions found by the PoseView interaction model
**5a**	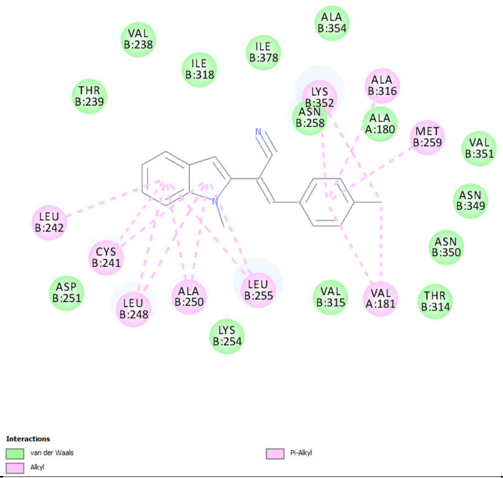	No interactions found by the PoseView interaction model
**5b**	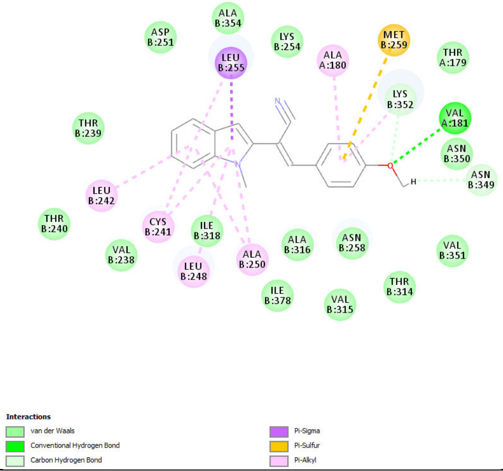	No interactions found by the PoseView interaction model

Table S4. Continued

Compd	Discovery Studio	PoseView
**5c**	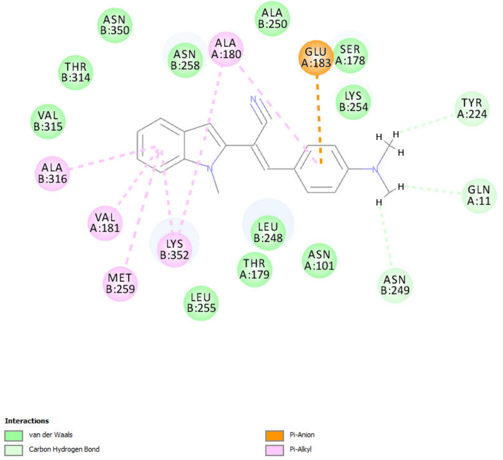	No interactions found by the PoseView interaction model
**5d**	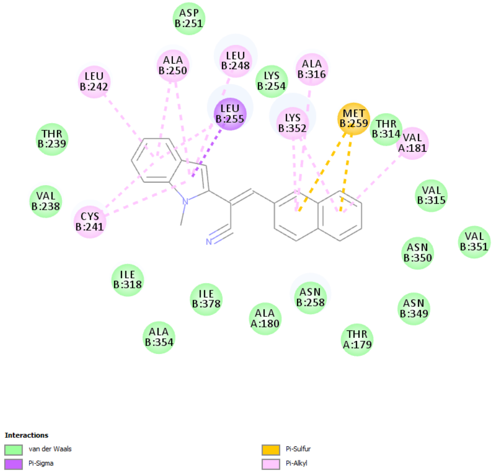	No interactions found by the PoseView interaction model

Table S5. The 2D diagrams of interactions created by compounds **2x** in the active site of penicillin-binding protein 4 (2ex8) generated by BIOVA Discovery Studio Visualizer and Pose View.

Compd	Discovery Studio	PoseView
**2x**	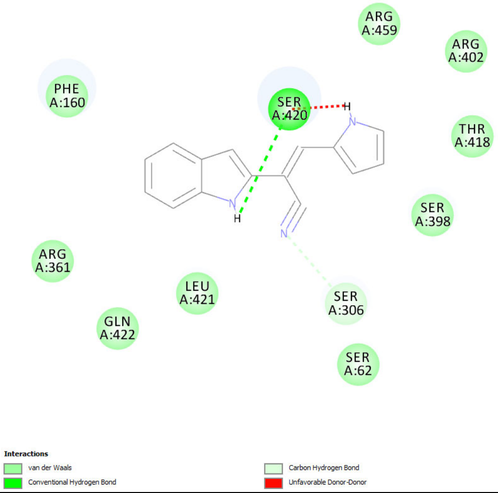	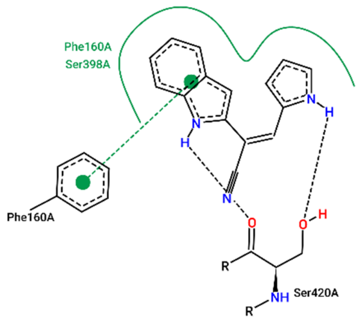

Table S6. The 2D diagrams of interactions created by compounds **2x** in the active site of β-lactamase (1fqg) generated by BIOVA Discovery Studio Visualizer and Pose View.

Compd	Discovery Studio	PoseView
**2x**	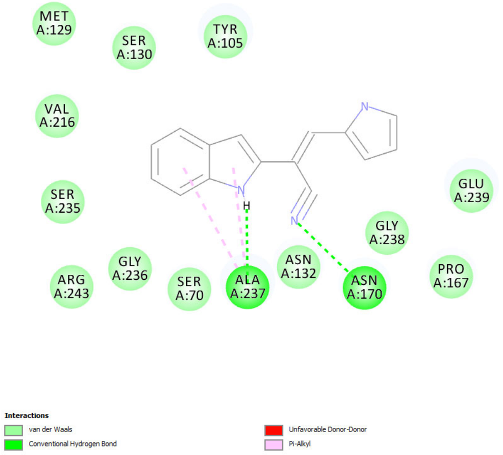	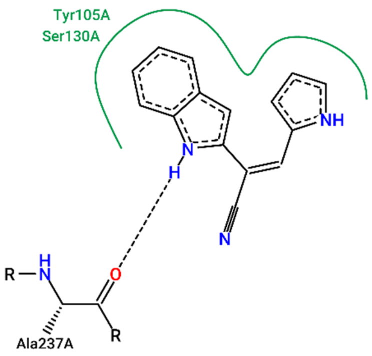

Tables S7. Predicted physicochemical, pharmacokinetic and drug-likeness properties of compounds **2l**, **2x**, **5a-d.**

	Molecule	2l	2x	5a	5b	5c	5d
**Physicochemical Properties**	Formula	$C_{19}H_{17}N_3$	$C_{15}H_{11}N_3$	$C_{19}H_{16}N2$	$C_{19}H_{16}N_2O$	$C_{20}H_{19}N3$	$C_{22}H_{16}N_2$
	MW	287.36	233.27	272.34	288.34	301.38	308.38
	Heavy atoms	22	18	21	22	23	24
	Aromatic heavy atoms	15	14	15	15	15	19
	Fraction Csp3	0.11	0.00	0.11	0.11	0.15	0.05
	Rotatable bonds	3	2	2	3	3	2
	H-bond acceptors	1	1	1	2	1	1
	H-bond donors	1	2	0	0	0	0
	MR	92.43	72.58	88.09	89.62	97.33	100.63
	TPSA	42.82	55.37	28.72	37.95	31.96	28.72
**Lipophilicity**	iLOGP	2.71	1.67	3.11	3.14	3.15	3.16
	XLOGP3	3.99	2.69	4.18	3.79	3.94	5.07
	WLOGP	4.19	3.45	4.44	4.14	4.20	5.29
	MLOGP	2.59	1.39	3.20	2.59	2.82	3.69
	Silicos-IT Log P	3.99	3.86	4.31	3.84	3.47	4.86
	Consensus Log P	3.49	2.61	3.83	3.50	3.51	4.41
**Water Solubility**	ESOL Log S	-4.44	-3.42	-4.56	-4.32	-4.48	-5.40
	ESOL Solubility (mg/ml)	1.04e-02	8.78e-02	7.53e-03	1.37e-02	1.01e-02	1.23e-03
	ESOL Solubility (mol/l)	3.62e-05	3.76e-04	2.76e-05	4.76e-05	3.35e-05	3.98e-06
	ESOL Class	Moderately soluble	Soluble	Moderately soluble	Moderately soluble	Moderately soluble	Moderately soluble
	Ali Log S	-4.59	-3.51	-4.49	-4.28	-4.31	-5.42
	Ali Solubility (mg/ml)	7.37e-03	7.29e-02	8.77e-03	1.51e-02	1.47e-02	1.18e-03
	Ali Solubility (mol/l)	2.57e-05	3.12e-04	3.22e-05	5.24e-05	4.89e-05	3.84e-06
	Ali Class	Moderately soluble	Soluble	Moderately soluble	Moderately soluble	Moderately soluble	Moderately soluble
	Silicos-IT LogSw	-6.08	-5.19	-5.92	-5.66	-5.64	-7.20

	Silicos-IT Solubility (mg/ml)	2.38e-04	1.49e-03	3.24e-04	6.31e-04	6.94e-04	1.95e-05
	Silicos-IT Solubility (mol/l)	8.28e-07	6.40e-06	1.19e-06	2.19e-06	2.30e-06	6.32e-08
	Silicos-IT class	Poorly soluble	Moderately soluble	Moderately soluble	Moderately soluble	Moderately soluble	Poorly soluble
**Pharmacokinetics**	GI absorption	High	High	High	High	High	High
	BBB permeant	Yes	Yes	Yes	Yes	Yes	Yes
	Pgp substrate	No	No	No	No	No	No
	CYP1A2 inhibitor	Yes	Yes	Yes	Yes	Yes	Yes
	CYP2C19 inhibitor	Yes	Yes	Yes	Yes	Yes	Yes
	CYP2C9 inhibitor	Yes	Yes	Yes	Yes	Yes	Yes
	CYP2D6 inhibitor	Yes	Yes	No	No	Yes	No
	CYP3A4 inhibitor	Yes	Yes	No	Yes	Yes	No
	log Kp (cm/s)	-5.22	-5.81	-4.99	-5.37	-5.34	-4.58
**Drug-likeness**	Lipinski violations	0	0	0	0	0	0
	Ghose violations	0	0	0	0	0	0
	Veber violations	0	0	0	0	0	0
	Egan violations	0	0	0	0	0	0
	Muegge violations	0	0	0	0	0	1
	Bioavailability Score	0.55	0.55	0.55	0.55	0.55	0.55
**Medicinal Chemistry**	PAINS alerts	1	0	0	0	1	0
	Brenk alerts	1	1	1	1	1	1
	Leadlikeness violations	1	1	1	1	1	1
	Synthetic Accessibility	2.52	2.32	2.57	2.51	2.70	2.66

Tables S8. Predicted human intestinal absorption, Caco-2 cell and MDCK cell permeabilities for compounds **2l**, **2x**, **5a-d.**

Compunds	Human intestinal absorption (%)	In vitro Caco-2 cell permeability (nm/s)	In vitro MDCK cell permeability (nm/s)
Rule	0 - 20 (poor)<br>20 – 70 (moderate)<br>70 – 100 (well)	<4 (low)<br>4-70 (moderate)<br>>70 (high)	<25 (low)<br>25-500 (moderate)<br>>500 (high)
2l	93.69	57.59	0.09
2x	90.15	12.20	48.88
5a	100.00	55.86	46.73
5b	98.27	55.43	14.80
5c	99.83	57.65	0.16
5d	100.00	55.74	54.34
